# Holistic approach to assess co-benefits of local climate mitigation in a hot humid region of Australia

**DOI:** 10.1038/s41598-020-71148-x

**Published:** 2020-08-26

**Authors:** Shamila Haddad, Riccardo Paolini, Giulia Ulpiani, Afroditi Synnefa, Gertrud Hatvani-Kovacs, Samira Garshasbi, Jonathan Fox, Konstantina Vasilakopoulou, Lawrence Nield, Mattheos Santamouris

**Affiliations:** 1 UNSW Built Environment, The University of New South Wales, Sydney, Australia; 2 Former Northern Territory Government Architect, Darwin, Australia

**Keywords:** Climate sciences, Climate change, Climate-change mitigation

## Abstract

Overheated outdoor environments adversely impact urban sustainability and livability. Urban areas are particularly affected by heat waves and global climate change, which is a serious threat due to increasing heat stress and thermal risk for residents. The tropical city of Darwin, Australia, for example, is especially susceptible to urban overheating that can kill inhabitants. Here, using a modeling platform supported by detailed measurements of meteorological data, we report the first quantified analysis of the urban microclimate and evaluate the impacts of heat mitigation technologies to decrease the ambient temperature in the city of Darwin. We present a holistic study that quantifies the benefits of city-scale heat mitigation to human health, energy consumption, and peak electricity demand. The best-performing mitigation scenario, which combines cool materials, shading, and greenery, reduces the peak ambient temperature by 2.7 °C and consequently decreases the peak electricity demand and the total annual cooling load by 2% and 7.2%, respectively. Further, the proposed heat mitigation approach can save 9.66 excess deaths per year per 100,000 people within the Darwin urban health district. Our results confirm the technological possibilities for urban heat mitigation, which serves as a strategy for mitigating the severity of cumulative threats to urban sustainability.

## Introduction

Urban areas face several challenges, including increased energy and resources consumption, health risks and vulnerability to extreme events^1^, that must be counteracted by structures and processes that advance the well-being of people and the planet to ensure the sustainability of urban systems^2^. Additional man-made changes to local and regional climate^3^ and the localized effects of urbanization induce higher surface and air temperatures in cities compared to those in rural areas^4^, a phenomenon known as the urban heat island (UHI) effect. The magnitude of a UHI varies between 0.4 °C and 11 °C^5^ and is affected by synoptic weather conditions, the local morphology and structure of the city, urban materials, anthropogenic heat generation by human activities, and heat sinks^6^. This effect is further exacerbated by global climate change leading to more frequent heat waves^7,8^ and severe consequences for urban sustainability. UHI is documented in more than 400 major cities around the world^5,9^.

UHIs increase the cooling energy demand of buildings depending on the magnitude of the urban overheating, microclimate, building characteristics and performance of air conditioning systems. On average, UHIs increase the cooling loads of urban buildings by 13.1% compared to rural buildings reference demand^10^. Each degree of temperature rise results in a 0.45–4.6% increase in the peak electricity demand, which leads to an electricity penalty of 21 (± 10.4) W/°C/person^11^. On top of the effects of urbanization, climate change and market penetration of air conditioning will put further stress on urban energy systems. It is expected that the average cooling energy needs of residential and commercial buildings in 2050 will increase by 750% and 275%, respectively^12^. The most significant increases are predicted to occur in India^12^, where hot-humid conditions are prevalent.

Beyond energy consumption, local and global climate change pose a burden to public health considering the significant increases in mortality and hospitalization during heat wave days, especially for the most at-risk residents^13^. More frequent and longer heat wave events adversely affect vulnerable people with low socioeconomic status^14–17^ and elderly individuals who are more susceptible to heat^18,19^. Further, in tropical areas, the prevalence of infectious disease mortality is amplified by rainfall and temperature increases, and an increasing trend in heat-related cardio-respiratory mortality has also been documented in hot-humid areas^20^.

While the majority of heat-related deaths occur indoors, the increased risks of heat exhaustion and heat stroke will substantially limit outdoor physical activity and work^17,21^ and reduce workers’ productivity^22^ across wider spatial and temporal scales. Thus, thermal comfort in urban open spaces is vital to sustainable cities yet complex due to psychological adaptation^23^.

Among the most impacted areas are tropical cities, where high-humidity regimes reduce the mitigation potential of evaporative cooling^24^ and increase thermal discomfort because transpiration is impaired^25–27^.

Climate change is worsening the situation. Over the last few decades, Australian cities have experienced longer, more intense and more frequent extreme heat events, which cause 55% of all deaths related to natural disasters^28^. By 2070, the Australia-wide annual average warming is expected to be between 2.2 °C and 5 °C^29^, and annual net temperature-related deaths will rise to 1,250 per year in the unmitigated climate change scenario^30^. In the tropical city of Darwin, Northern Territory, residents could be subjected to up to 227 days per annum above 35 °C by the year 2070, with an annual average temperature of 31 °C^31^. The heat released by widespread use of air conditioning^32^ and man-made materials such as asphalt pavements^33^ exacerbates the heat wave conditions in hot-humid regions.

Extensively investigated local climate mitigation technologies^34,35^ have the potential to reduce average peak ambient temperatures by 2–3 °C^36,37^. However, the body of knowledge focusing on the overheating conditions and cooling potentials in tropical cities analyses different mitigation strategies^38^, while a quantification of the most relevant co-benefits and their interrelation is still needed. More in detail, little research has been conducted—especially in hot-humid regions—to quantify the potential of these heat mitigation measures to reduce energy consumption, reduce peak electricity demand and improve health using an integrated and holistic approach.

The synergy among energy, health, comfort, and vulnerability emphasizes the need to apply an integrated interdisciplinary framework for urban sustainability research. In the context of sustainable urban systems, we present a new approach to counteract urban overheating yielding sustainability outcomes both locally and globally. Using extensive measurements of local and urban climate, we analyze the characteristics of urban overheating in the hot-humid city of Darwin. We also analyze urban heat mitigation measures using a microclimate simulation tool and evaluate the impacts of several changes to urban cover and the urban fabric on building cooling energy needs, peak electricity demand and health.

### Local urban and suburban climate

**Figure 1:**
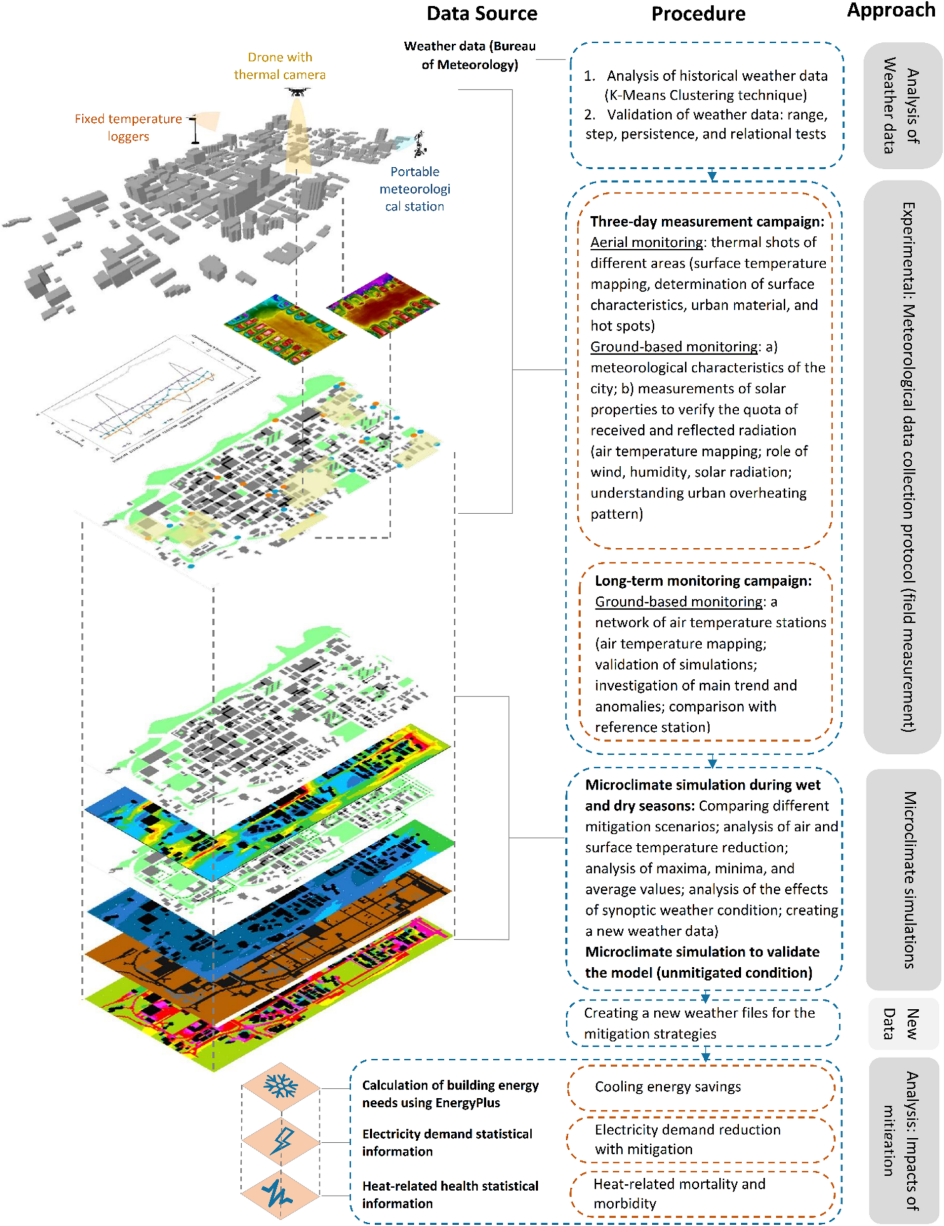
Main methodological approaches and framework used in this study.

We identified four main methodological approaches namely statistical analysis of climatic data, experimental campaigns, microclimate simulation, and analysis of the heat mitigation impacts (Fig. 1), applied either separately or in combination.

In all clusters of climatic data analyzed for a 10-year period, the air temperature exceeded 36.0 °C, and the average relative humidity (RH) varied from 66 to 77% (see “Methods” and Supplementary Figure 1). The highest ambient temperatures (with an average of 29.4 °C) occurred in 24% of the year, combined with a northwesterly wind direction and an average wind speed of 4.7 m/s. The lowest average ambient temperature (high average humidity) is observed during the periods when wind directions were from southeastern and north to northeastern direction which is likely influenced by the national parks and development of a sea breeze flow. Southerly and southeasterly wind directions were the prevailing synoptic conditions for the minimum ambient temperature and humidity levels, with an average air temperature, RH, and wind speed of 25.3 °C, 66% and 4.1 m/s, respectively.

Once the synoptic boundary conditions were delineated, we carried out a short-term (three days) monitoring to get an evidence-based understanding of how the heat island developed and distributed throughout the cityscape, with due attention to the city hot and cool spots. We measured the local microclimatic conditions and thermal response of the materials to strike links between potential causes of and remedies to urban overheating and substantiate the selection of the mitigation scenarios (Supplementary Figure 2). Our short-term ground-based monitoring confirmed a significant difference in the spatial distribution of air temperature (T_a_) at the pedestrian level, with the maximum T_a_ difference between urban and suburban areas, ΔT(_urban-suburban_), exceeding 2.0 °C. The average wind speed decreased to 0.5 m/s in the urban area, 10 times lower than that recorded at the suburban station. Since the cityscape in Darwin is dominated by low-rise buildings and relatively wide streets, the wind breaking was attributed to a strong convergence induced by the heat island circulation^39^. The RH was higher at all urban measurement points, exceeding 75%. The spatial distribution of T_s_ based on the data obtained during short-term ground-based monitoring are presented in Supplementary Figure 3. Our infrared aerial monitoring recorded very high surface temperatures (T_s_) for the streets of Darwin, exceeding 60 °C. Bitumen surfaces in parking lots reached temperatures of 67 °C. Roofs and street pavements also recorded high T_s_ values, with peak temperatures of 66 °C and 56 °C, respectively. Dark, low-albedo construction materials used in the urban fabric potentially affect local climatic conditions by absorbing incoming shortwave radiation and reradiating it as longwave thermal radiation. Trees recorded lower T_s_ values (below 38 °C) and reduced the T_s_ values of streets and pavements by over 10 °C through shading.

**Figure 2:**
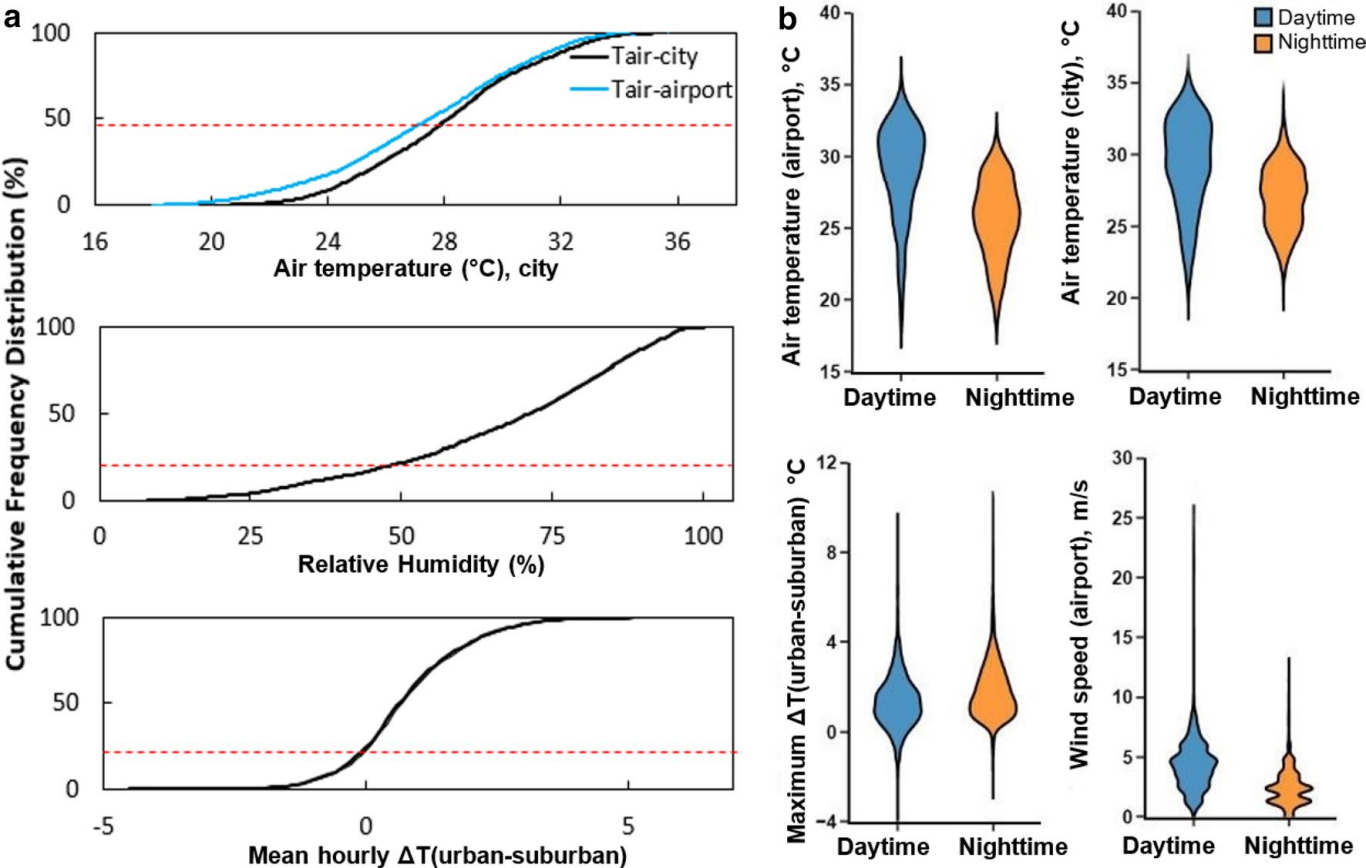
Cumulative frequency distribution of T_a_, relative humidity and hourly ΔT(_urban-suburban_) over a year (**a**), box plots of T_a_, wind speed and maximum ΔT(_urban-suburban_) (**b**).

A second, long-term, monitoring campaign was later arranged to expand the investigation during both dry and wet season and get an understanding of the year-round variability and spatial heterogeneity of the heat island distribution. The long-term monitoring campaign documented consistent warm and humid conditions throughout the year, with the 50th percentile of hourly T_a_ above 28 °C, while the absolute minimum temperature was approximately 20.0 °C (Fig. 2). The hourly ΔT_(urban-suburban_) was positive for 95% of the studied period and surpassed 5.0 °C. ΔT_(urban-suburban_) was larger at lower wind speeds and declined as wind speeds increased, highlighting the role of advection in urban cooling. The nocturnal mean T_a_ was consistently higher in the city (on average 1.3 °C higher) than in the suburban area, suggesting that the city of Darwin cools less rapidly than its surroundings (Supplementary Figure 4).

During the study period, the RH was above 60% for more than two-thirds of the year. The calculated wet-bulb globe temperature (WBGT) was above 28 °C and 30 °C for 75% and 54% of the time, respectively, raising heat stress concerns during strong and extended physical exertion (Supplementary Figure 6). Similarly, the heat index showed that residents were safe from heat in the shade for only up to 23% of the time, and exposure to direct solar radiation would potentially increase the risk of dangerous heat disorders such as hyperthermia.

Darwin City Central suffers excessively from overheating primarily because of the way it is built. As detailed in Supplementary Table 4, 35% of the city of Darwin is covered by unshaded blacktop bitumen and over 80% of pathways are made of concrete and aggregate. Monitoring campaigns suggest that the overheating is amplified by the high T_s_ values of the urban fabric that increase T_a_, urban morphology that traps heat within the urban canopy, reduced wind speeds and lack of cooling sea breeze due to elevated sea surface temperature (SST). The advection rate in the city of Darwin depends on the urban form and layout of urban canyons. Areas with low-rise open layouts recorded lower daytime ambient temperatures than other parts of the city. The cooling effects of urban green spaces were also apparent in our results (Supplementary Figure 5). Further, locally released anthropogenic heat from air conditioning additionally increases local temperatures in urban areas^40,41^. However, our campaign did not quantify its relative weight. Thus, quantification of anthropogenic heat, for instance by means of a combination of inventories and eddy covariance measurements, is required to assess in detail its relative contribution to Darwin’s local climate.

### Mitigation potential of the proposed strategies

**Figure 3:**
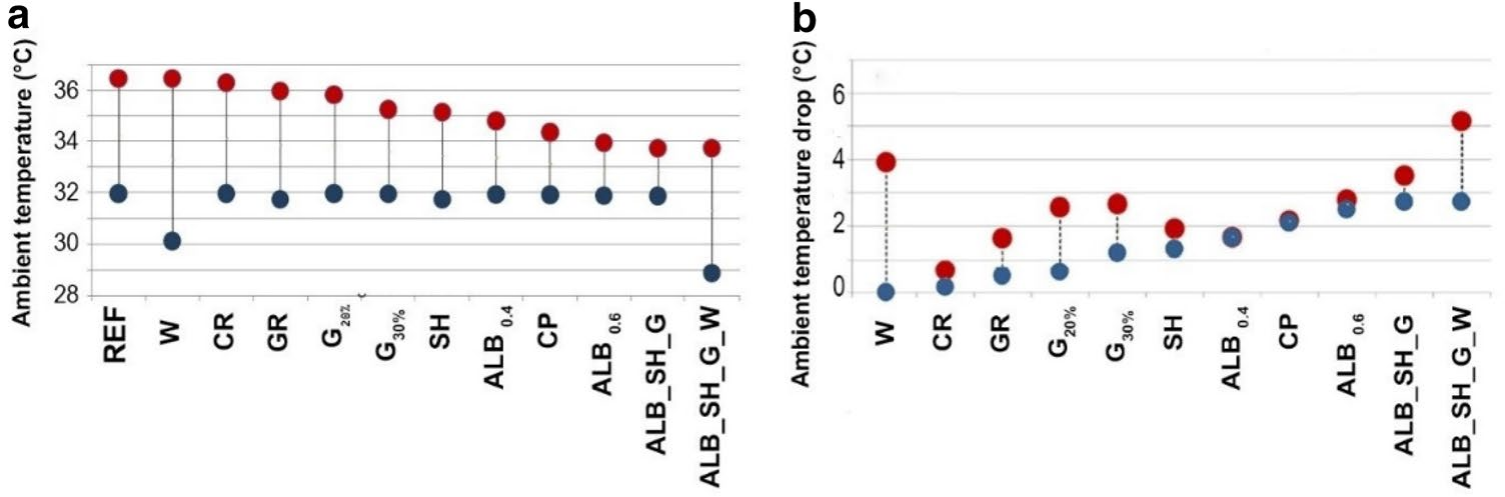
The range of ambient temperature and air temperature reduction: T_a,MAX_ (red marker) and T_a,MIN_ (blue marker) (**a**), ΔT_a,MAX_ (°C) (blue marker) and the MAX ΔT_a_ (red marker) (**b**). REF (unmitigated), ALB_SH_G (Albedo, greenery and shading), ALB_SH_G_W (albedo, shading, greenery and water), SH (shading), CP (cool pavement), ALB 40% (albedo 0.4), ALB 60% (albedo 0.6), CR (cool roof), G 20% (greenery 20%), G 30% (greenery 30%), W (water), GR (green roof).

We examined 11 mitigation scenarios for decreasing the ambient temperature by the increase of greenery, application of cool materials, water spray system, shading, green roof, and combination of the selected strategies (see “Methods”). The results of microclimate simulations are discussed here for the peak hour (14:00) of a typical warm day (20th February 2016) during wet season. In the simulated unmitigated scenario, T_a_ varies between 32.0 °C and 36.4 °C, and T_s_ exceeds 55 °C (Fig. 3).

**Figure 4:**
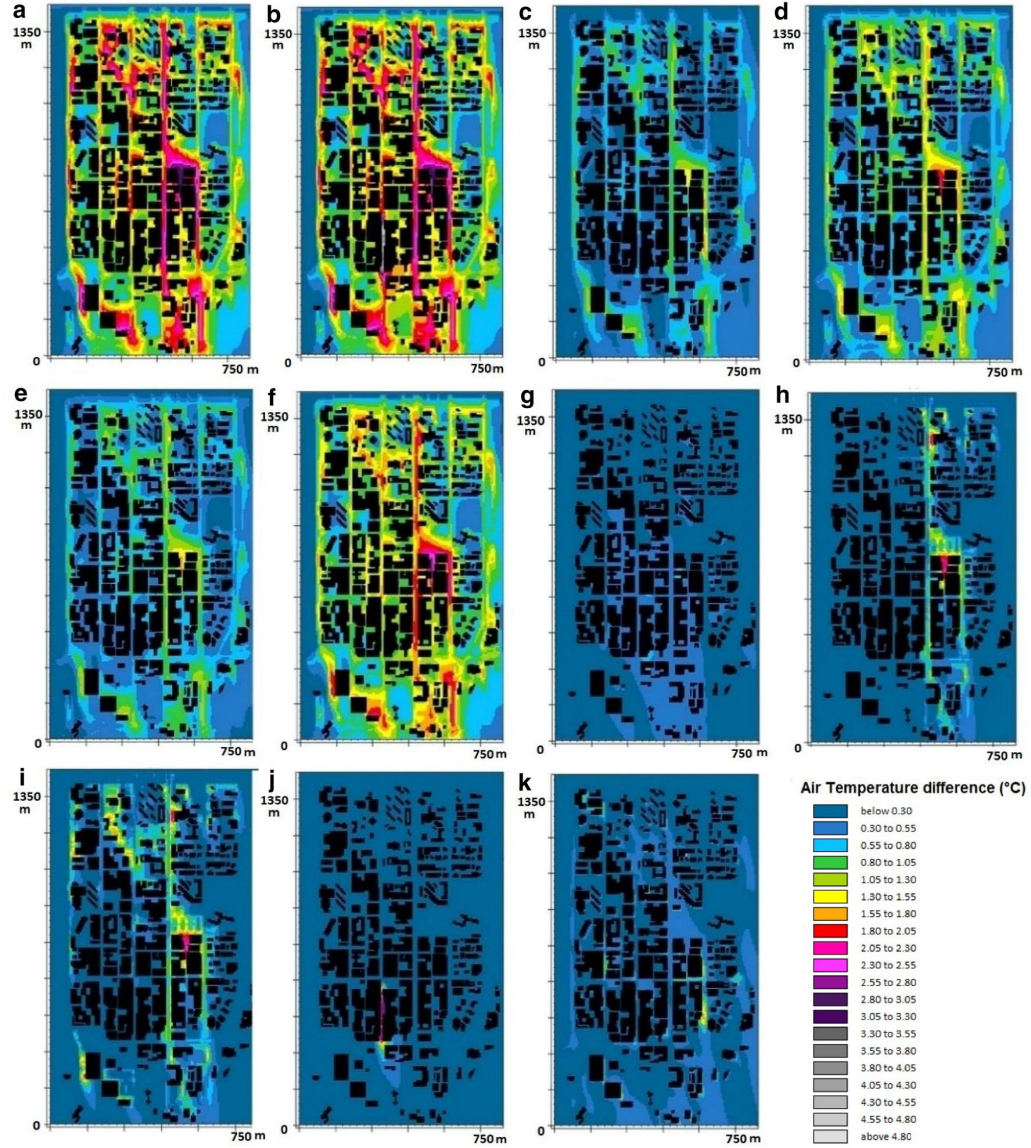
Spatial distribution of T_a_ in each mitigation scenario against that in the reference condition under the prevailing summer condition: ALB_SH_G (**a**), ALB_SH_G_W (**b**), SH (**c**), CP (**d**), ALB 40% (**e**), ALB 60% (**f**), CR (**g**), G 20% (**h**), G 30% (**i**), W (**j**), GR (**k**).

Simulation results (Fig. 4, Supplementary Table 10) demonstrate maximum ΔT_a_ values of 1.7 °C and 2.8 °C for precinct-scale increases in albedo of 0.4 and 0.6 (ALB_0.4_ and ALB_0.6_), respectively. The peak surface temperature (T_s,MAX_) is reduced by 11 °C, and the highest reduction in surface temperature (MAX ∆T_s_) reaches 15 °C. The scenario with cool pavements (CP) shows air temperature range of 31.9–34.3 °C and MAX ∆T_a_ and MAX ∆T_s_ values of 2.2 °C and 15.8 °C, respectively. T_a_ ranges from 31.7 to 35.1 °C when shading is used in the main streets (Fig. 3). Solar control reduces T_a,MAX_ by 1.3 °C and decreases T_s,MAX_ by 12 °C. The results demonstrate that increasing greenery coverage (G_20%_ and G_30%_) could trim T_a,MAX_ by 1.2 °C and lower T_s_ by 11–23 °C. This results in a MAX ∆T_a_ value of approximately 2.6 °C. By increasing the albedo of all building roofs (CR), T_a,MAX_ is reduced by 0.2 °C, and MAX ΔT_a_ reaches 0.7 °C. CR results in the pedestrian-level T_a_ ranging from 32 to 36.3 °C, which indicates a negligible impact on reducing ΔT_s,MAX_. The application of green roofs (GR) on all buildings reduces T_a,MAX_ and T_s,MAX_ by 0.5 °C and 2 °C, respectively, and results in a MAX ∆T_a_ value of 1.6 °C. The use of 10 evaporative cooling systems locally in the central pedestrian mall (W) leads to a significant local ambient temperature drop of 3.9 °C (Fig. 3). The combined mitigation scenario (ALB_SH_G) reduces T_a_ by 2.7 °C and T_s_ by 26 °C. By adding evaporative cooling systems, the combined scenario (ALB_SH_G_W) indicates a MAX ΔT_a_ value of 5.2 °C in areas where water spray systems were used.

**Figure 5:**
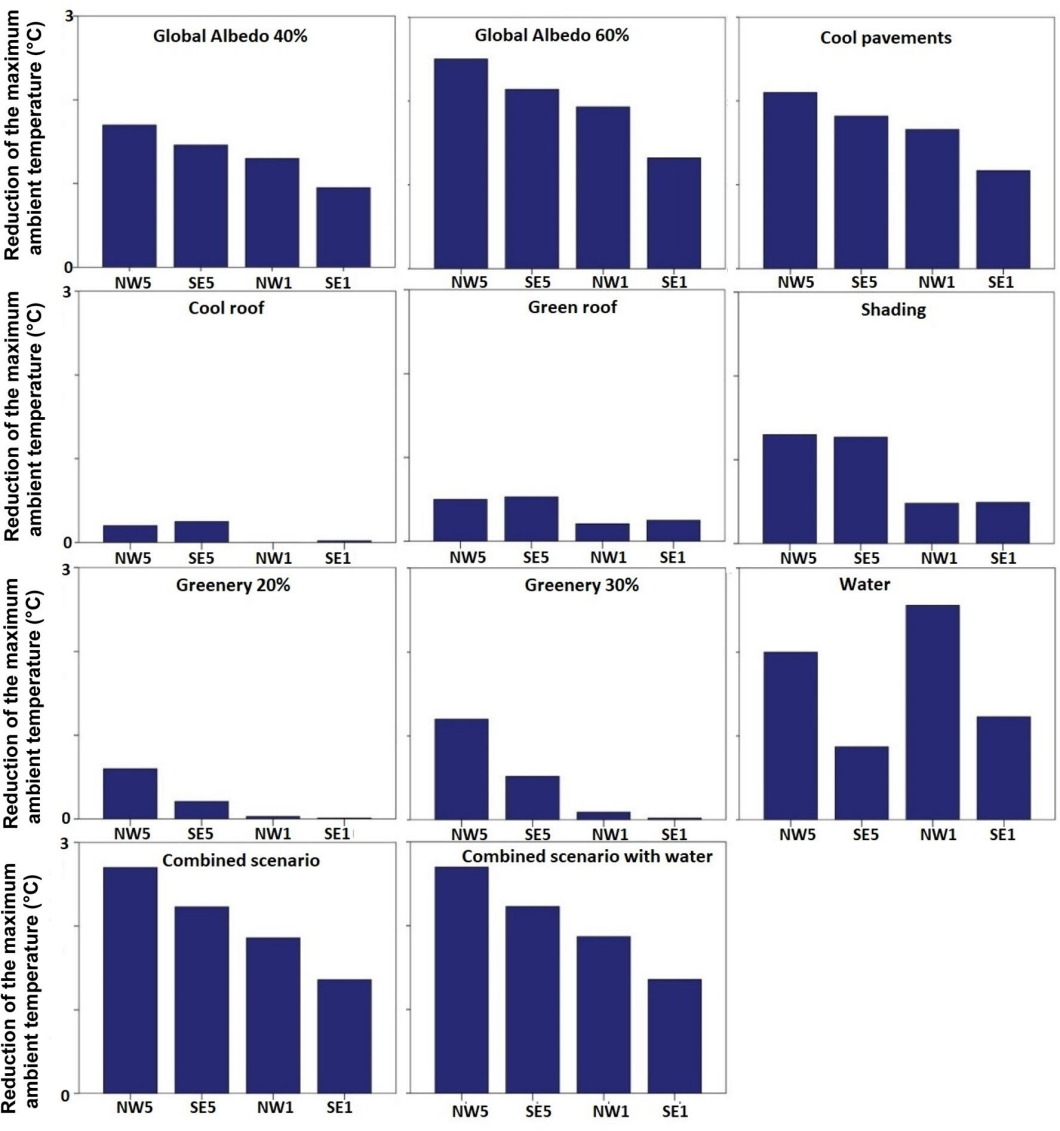
∆T_a_, _MAX_ calculated under NW5 = North-Westerly wind of 5 m/s, SE5 = South-Easterly winds of 5 m/s, NW1 = North-Westerly wind of 1 m/s, SE1 = South-Easterly winds of 1 m/s.

The mitigation potential of the strategies varies considerably as a function of synoptic weather conditions (Fig. 5). The use of CP, SH, G, and their combination shows higher ambient temperature reduction under higher wind speeds, while the cooling effect of water misting on air temperature decreases under higher wind speeds. The wind direction contributes to the MAX ∆T_a_ values in the G and W scenarios being 40–50% higher under a northwesterly wind direction.

### Impacts and benefits of mitigation technologies

We evaluated the impacts of the unmitigated and mitigated local climate profiles on cooling energy needs, electricity demand, and heat-related mortality and morbidity rates during the wet and dry seasons in 2016 using three selected mitigation strategies: i.e., urban greenery, cool material, and combination of selected strategies (Supplementary Table 11).

#### Cooling energy savings

**Figure 6:**
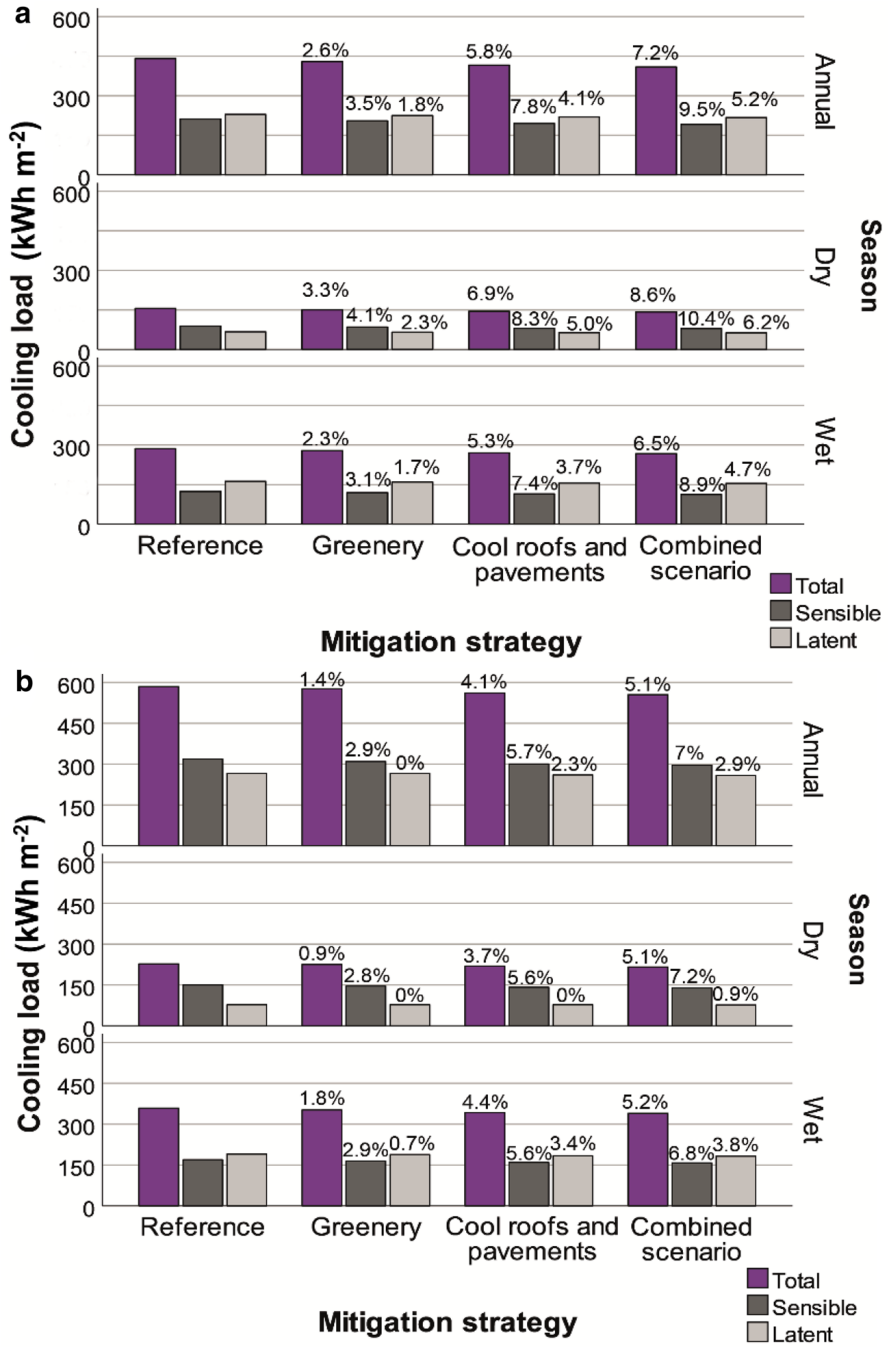
Cooling load of the residential (**a**) and office (**b**) buildings.

The calculated annual total cooling load is 441 kWh m^−2^ (Fig. 6a) for residential buildings in the unmitigated condition. Urban greenery reduces the annual cooling load of residential buildings by 11.6 kWh m^−2^ (2.6%); cool roofs and pavements by 25.8 kWh m^−2^ (5.8%); and the combination of greenery, cool roofs and pavements and urban shading by 31.9 kWh m^−2^ (7.2%). Cooling load savings in the wet season are higher (by 22–28%) than those in the dry weather regime, consistent with the higher air temperature reduction observed during the wet season. For office buildings, the calculated annual total cooling load is 585 kWh m^−2^ in the unmitigated condition (Fig. 6b). Urban greenery can reduce the annual cooling load of office buildings by 8.3 kWh m^−2^ (1.4%); cool roofs and pavements by 24.3 kWh m^−2^ (4.1%); and the combined scenario by 30 kWh m^−2^ (5.1%). The cooling load savings in the wet season are higher (by 37–67%) than those in the dry season.

Cool materials and urban greenery may result in residential cooling load savings of 137.2 GWh and 62.0 GWh, respectively, which are 19% and 64% lower than the cooling load savings achieved by the combined scenario (170.1 GWh). The combined scenario saves 95.1 GWh in commercial buildings, while cool materials and greenery lead to savings of 76.8 GWh and 26.4 GWh, respectively. On average, 68% of the annual cooling load savings occur during the wet season, and the remaining 32% occur during the dry season. The annual total cooling load savings for residential and commercial buildings resulting from the application of greenery and cool materials in the city of Darwin are estimated to be 88.4 GWh and 214 GWh, respectively. The cooling load savings for these building stocks exceed 265 GWh y^−1^ with the combinations of strategies, which is 67% and 19% more than that for greenery and cool materials, respectively.

#### Electricity demand reduction with mitigation

**Figure 7:**
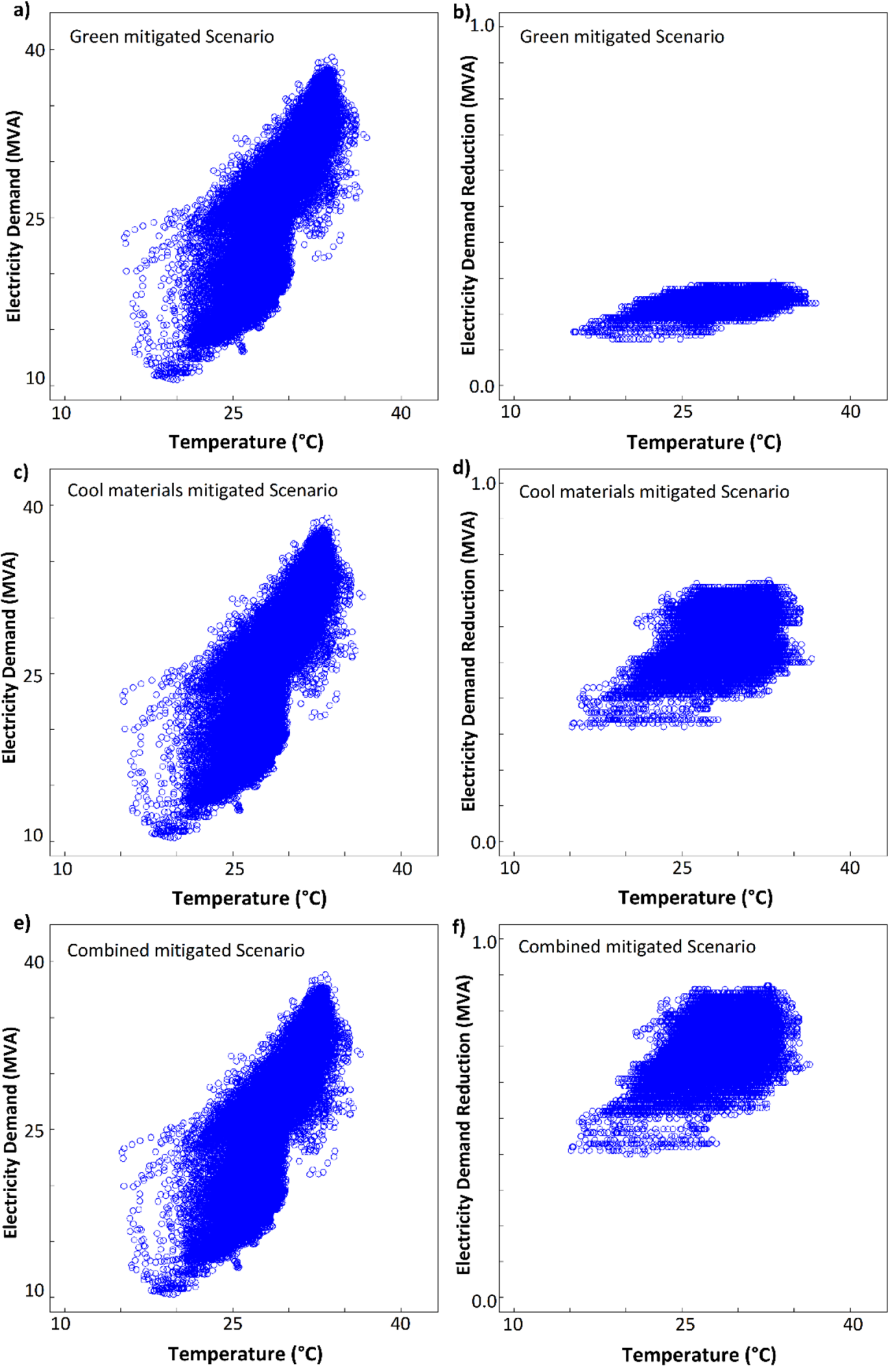
Electricity demand and absolute savings in the green mitigation scenario (**a**,**b**), cool materials scenario (**c**,**d**) and combined scenario (**e**,**f**).

All mitigation strategies reduce the electricity demand because the ambient temperature exceeds 18 °C, which is the temperature of minimum demand, for most of the year^42^ (the electricity demand data account for all consumption sources, not limited to air conditioning). The use of greenery offers only a modest reduction with peak savings of 0.3 MVA, while the combination of mitigation strategies achieves the largest savings of 0.8 MVA (Fig. 7, Supplementary Table 12). This corresponds to a peak electricity demand reduction of 2% compared to the unmitigated scenario.

#### Heat-related mortality and morbidity

The anomalies in hospital admissions were calculated based on the relation found with the daily maximum and mean temperatures, where anomalies in hospital admissions increased by 7% and 8% for every 1 °C increase in daily maximum and mean temperatures, respectively. The anomalies in daily deaths within the whole population were estimated based on the regression model developed, where an increase of 1 °C in daily maximum temperatures raises the medians and averages of the mortality anomalies by 4.9 and 4.4%, respectively. Last, the model from Woodruff et al.^79,80^ was used to calculate the estimated number of heat-related mortality among people with age of 65 and above.

The annual anomalies in hospital admissions and deaths were estimated per 100,000 population. The application of cool materials and greenery reduces the annual excess hospital admissions of 40.14 to 27.51 and 34.67, respectively. The combined scenario decreases the annual excess hospital admissions to 24.49 per 100,000 population. During the days with maximum air temperatures and humidity levels above the 80th percentiles, the estimated 0.96 excess hospital admissions in the unmitigated condition is reduced to − 12.21 with the combination of strategies. These results reveal the temperature and humidity impacts on health^43^ and highlight the positive contribution of the mitigation strategies.

**Figure 8:**
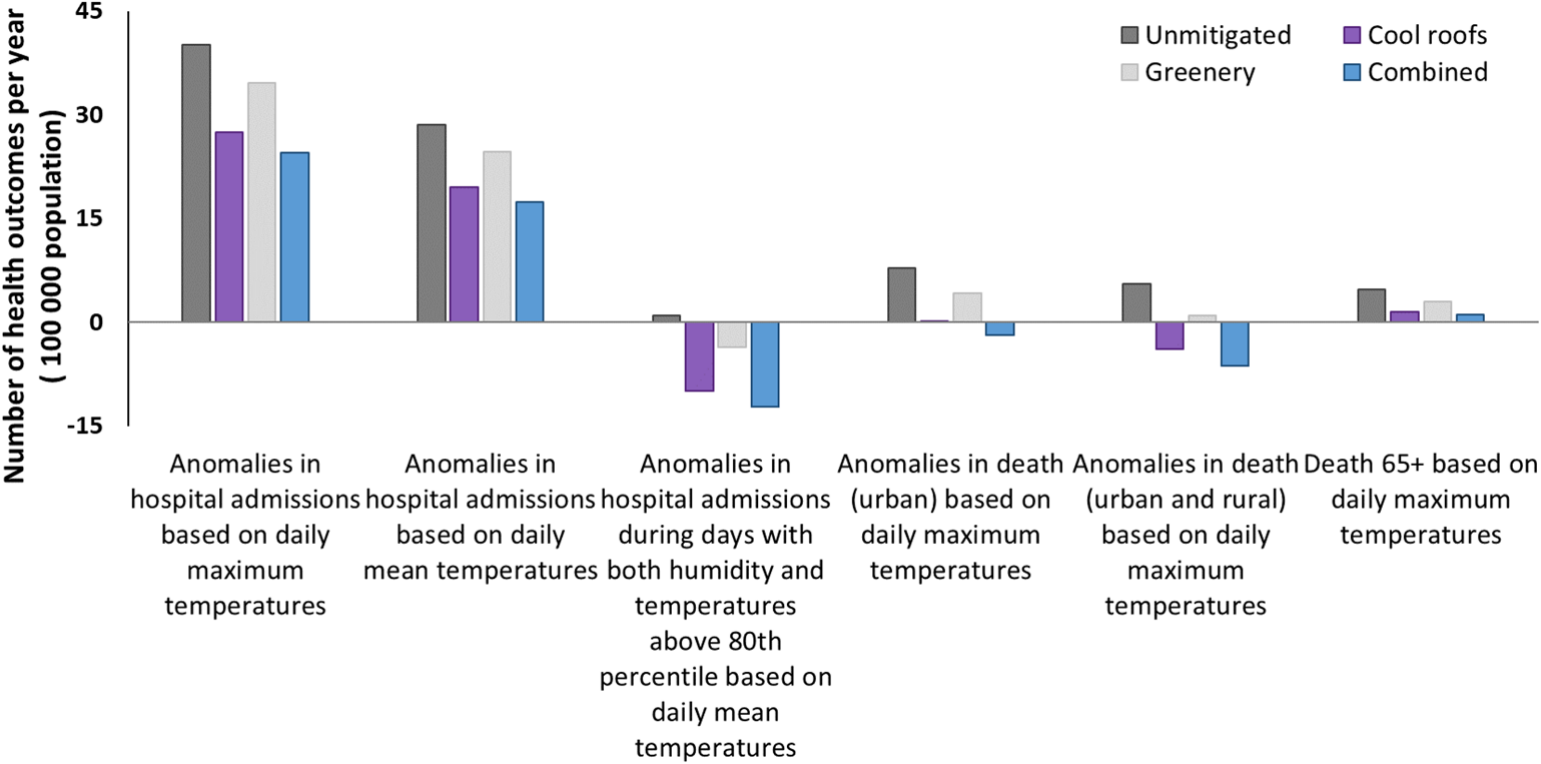
Calculated impact on the annual cumulated anomalies in morbidity and mortality per 100,000 population in the unmitigated and the mitigated scenarios the Darwin Urban Health District, and the Urban and Rural Health Districts.

The combination of SH, G and CP saves 9.66 excess deaths per year per 100,000 people within the Darwin urban health district based on our developed model (Fig. 8). The number of saved lives reaches 12 when both the urban and rural health districts are considered. Four deaths can be avoided per annum per 100,000 people aged over 65 with the implementation of the combined mitigation scenario (Fig. 8).

## Discussion

Managing the adverse effects of urban overheating needs a holistic interdisciplinary approach to understand how the urban environment is built and responds to climate. This study offers an evidence-based methodological approach to fill the existing knowledge gap through a comprehensive assessment of the impact of mitigation technologies in energy, peak electricity demand, mortality and morbidity. We evaluated the impacts of mitigation technologies applied in the tropical city of Darwin, which is particularly susceptible to heat-related illness with increasing temperature due to global and local climate change.

The city of Darwin experienced 1,764 h above 30 °C during the period of this study (2017–2018). The combination of high ambient air temperatures and high RH levels presents a substantial challenge for human thermoregulation^44^. This is particularly relevant to labor-intensive outdoor work. The magnitude of urban overheating in Darwin is affected by several factors: the use of materials with high solar absorptance in the urban fabric that stimulate advection and thermal radiation; reduced wind speed in the urban area and lack of sea breeze due to insufficient thermal gradient between land and sea; and release of anthropogenic heat into the city by air conditioning units and traffic that increases the urban ambient temperature by 1 ºC^45^. In this study, we have demonstrated that the implementation of heat mitigation strategies can measurably slow the rate at which the city of Darwin is warming.

The cooling potential of the mitigation strategies varies as a function of background synoptic conditions. Increased wind speeds have a positive impact on the cooling potential of reflective surfaces, shading, and greenery due to the increased convective cooling of the built surfaces and evapotranspiration potential of urban greenery. In contrast, higher wind speeds decrease the mitigation potential of water spray systems because of accelerated removal of water droplets from the target area. In this study, we used mist cooling as an efficient mitigation strategy in hot-humid climates, as advocated in recent studies^46^. By pulverizing water into fine droplets, these systems not only use an extremely modest water flow rate (further reduced when control logics are in place^47^) but also let heat transfer (evaporation) occur even in very humid air because of the high surface-to-volume ratio.

The urban form and layout in the city of Darwin affect the advection and thus change the cooling potential of the strategies under different synoptic conditions. Local and global application of mitigation strategies also impact cooling potential. The mitigation technologies contribute to decreasing T_s_ and thus improving outdoor thermal comfort by reducing the emitted infrared radiation and convected sensible heat from opaque surfaces.

The ambient temperature in Darwin, which has a tropical savanna climate^48^, exceeds the typical setpoint for cooling (here 24 °C) for approximately 90% of the time, which highlights the important cooling demand of buildings. A large fraction of the cooling loads in Darwin are represented by latent heat loads due to the high humidity levels for substantial periods of the year. Thus, the application of mitigation techniques has a negligible impact on the latent cooling demand. Regarding the sensible load, we obtained a decrease of approximately 10% (25–30 kWh m^−2^ y^−1^) with the mitigation strategies. The maximum total cooling load reduction with the most effective mitigation strategy is 7.2% and 5.1% for residential and office buildings, respectively. Although the reduction percentage may seem low, the impact of the mitigation strategies results in large cooling demand savings based on the absolute values (30–32 kWh m^−2^ y^−1^).

To achieve greater reductions in residential and commercial building energy needs, the focus should be placed on improving building energy performance. We considered uninsulated buildings with poor thermal performance representing building stocks developed prior to the introduction of building energy efficiency regulations. However, the cumulative annual savings in the combined scenario account for more than 250 GWh. Natural ventilation is not a viable option to maintain comfortable indoor conditions due to the high ambient humidity and lack of cool sea breeze to reduce the ambient temperature during nighttime. This is a challenge in many hot-humid areas where sea breezes bring no relief because of high SST and limited air–sea interactions. Uncertainty in the absolute values of cooling energy savings at the urban scale may be expected^49^ due to the diversity of building stocks and the variance in the adaptive behavior of building occupants^50^. Our results are presented relative to the unmitigated scenario; nevertheless, we acknowledge high nonlinearity in user behavior, such as opening windows^51^.

The performance of the surrogate model is robust for the peak and minimum electricity demand; however, it is suboptimal in the intermediate demand range. The peak electricity demand is approximately 5.5 kW per capita for the population of over 7,000 people in the city of Darwin. The peak demand reduction is 0.11 kW, which is not negligible in absolute terms. The city of Darwin has a small area with several hotels and a rapidly expanding population, inducing high uncertainty in the estimation of individual energy use. Therefore, it is not possible to normalize the demand per capita to compare different periods for significant changes to population density and use long data series.

The absolute humidity has a nonlinear effect on the electricity demand. This is likely due to the dehumidification process in traditional air conditioning systems, which need a coil temperature lower than the dew point temperature to remove moisture by condensation. After moisture removal, the air is reheated to supply comfort conditions, which generates an excess load. Other types of building cooling technologies, such as desiccant or hybrid air conditioning, offer potential alternative solutions for overcoming this issue^52^.

Exposure to hot-humid conditions outdoors and physical exertion lead to the development of heat stress or stroke due to the limited potential for body heat loss, hydration, and shaded rest breaks that reduce the accumulation of body heat. Our mitigation scenarios decrease the negative heat-related health outcomes, and the largest improvement is achieved with the combination of strategies. Nevertheless, this study does not discuss the benefits of denser green infrastructure, such as reduced storm water run-off, air pollution reduction, increased space for recreation and higher biodiversity^53–55^.

Climate change threats to health reinforce the need for mitigation and adaptation of urban areas and buildings to reduce heat exposure and public health programs^17^. However, evidence-based approaches to sustainable development require integrated knowledge about the environment, development, and health^56^. Although the implications of research findings may vary depending on the context and time scale, a holistic methodological approach is crucial to shift toward a more environmentally sustainable future. Thus, it is recommended to address the challenges of energy sustainability and climate change through interdisciplinary and mission-oriented research coupled with the science of human and natural systems^57^.

## Methods

### The study area

Darwin is the capital of the Northern Territory (NT), located in the northernmost part of Australia. It features a tropical savanna (Aw) climate^48,58^. Darwin local government area (LGA) recorded 78,804 residents in 2016^59^. It covers a land area of 112 km^2^, and the total floor area is 8,492,553 m^2^ for residential and commercial buildings^60^. The monitoring and mitigation study covers a land area of about 1.0 km^2^, which is part of the Darwin City Central with an area of 3 km^2^. We assessed the impacts of the mitigation at different city scales based on the availability of the required data. We received the semi-hourly electricity demand data from Power and Water Corporation for the Darwin City Central, which comprises the City Centre and the Frances Bay area. We obtained land use data from Darwin LGA for building energy demand analysis. Darwin urban and rural health data were obtained from the department of Health and families for Greater Darwin (3,163 km^2^) and Darwin rural area.

### Weather data

Semi-hourly weather data were obtained from the Bureau of Meteorology (BoM) weather station located in Darwin airport (Lat: 12.42°S; Long: 130.89°E; elevation: 30.4 m above mean sea level, BoM station number 014015, WMO ID 94120), 6 km from the city, based on the records of the last 10 years from 2007^61^. The measured quantities are dry-bulb and wet-bulb air temperatures, wind speed and direction, and air pressure. Moreover, also 1-min radiation data are collected, including global horizontal irradiance, diffuse irradiance, and incoming thermal radiation. The location of the airport station is provided in Supplementary Figure 2. We performed an analysis of the hourly climate data recorded over 10 years at the Darwin BoM airport station and found four clusters of synoptic weather conditions for the three main climatic parameters: i.e., ambient temperature, ambient humidity and wind direction. The K-Means Clustering technique was used to classify the data and extract the main scientific conclusions. The analysis permits to understand the main climatic characteristics of the city (Supplementary Figure 1). Humidity and temperature are clustered in four groups of very high, high, average, and low conditions. The four clusters of wind directions are north to east (0°–90°), east to south (9°–190°), south to west (190°–290°), west to north (190°–360°).

For the purpose of the energy and electricity demand analysis, we focused on semi-hourly data obtained from February 2016 to December 2017. Unrealistic values were filtered by performing range, step, persistence, and relational tests^48^. The validation procedure for the radiation data was performed on the 1-min raw data from Darwin airport weather station (part of the Baseline Surface Radiation Network^62^) by the same procedure described above. We used the solar position calculated with the model by Reda and Andreas^63^ to cut off nighttime solar readings (due to instruments zero offset) and linearly interpolate with the cosine of solar zenith angle for missing values.

To fill in the gaps in the data series, we linearly interpolated between values for gaps of three hours. To establish a relation between the measurement campaigns in the city of Darwin and the airport, we used the genetic algorithms implemented in the heuristic software tool Eureqa^64^. With the developed correlation, we computed the urban air temperatures in the unmitigated scenario and then computed the mitigated ambient temperatures using the correlations found for the dry (May to October) and wet (November to April) periods.

### Data collection protocol

#### Three-day measurement campaign

We performed the measurement campaign during the wet season from 23rd to 25th March 2017. The aim of short-term monitoring was to identify the distribution of the urban microclimate in the city by collecting aerial thermal images of the urban surface and ground-based microclimatic parameters through a mobile sensor network. Local specificities (e.g. materials, canopy, proximity to the sea) were thus linked to the onset of hot and cool spots to substantiate the design of bespoke mitigation scenarios. The resulting database provided the necessary information for the first simulation run in the wet season and was used to compare the patterns of the measured and simulated surface temperatures. Supplementary Tables 1 to 3 show the equipment specifications.

##### Aerial monitoring

The ground-based monitoring was performed on 23rd and 24th March 2017. We recorded the surface temperatures of the materials used in the urban fabric by aerial infrared (IR) imaging with a drone. The thermal camera collected images and videos to capture extensive, time-aligned visual information on surface temperature consistent with recent studies^65,66^, with the thermal emittance of typical values for material classes. It was complemented with the reflectance properties retrieved by a ground-level net radiometer.

##### Ground-based monitoring

The ground-based monitoring was performed on 24th and 25th March 2017 from 11:00 to 18:00. We collected air temperature, RH, wind speed and direction, solar radiation and heat flux at 20 points (Supplementary Figure 2). Measurements were taken at 1.1 m above the ground representing the height of a standing person^67^. The net radiometer was located at 0.5 m above street level to record ground-level radiative data and optical properties. The analogic outputs of the net radiometer and the serial SDI-12 outputs of the weather station were connected to a datalogger (DataTaker DT85). The monitoring methods were developed based on existing literature^5,68–74^.

#### Long-term monitoring campaign

We carried out measurements of air temperature over twelve months from 11th September 2017. The aim was to depict the year-round dynamical modifications of the heat island distribution across the city, accounting for dry and wet season specificities. The long-term data collection informed a second simulation run, with extensive time and space-resolved calibration. The resulting database allowed a solid validation of the model. The ambient temperature was simultaneously recorded every 30 min in 15 locations in the city of Darwin using LogTag TRIX-16 temperature recorders (Supplementary Figure 2). Each temperature recorder was placed in radiation shields and mounted to the streetlight poles at a height of approximately 2.5 m above the ground. The obtained data were analyzed and compared with a reference located in Darwin airport to determine ΔT_(urban-suburban)_.

### Microclimate simulations

We simulated the microclimate and the environmental conditions in Darwin with the software model ENVI-met V4.1.2. This 3-D microclimate model simulates the all-wave radiative (solar and infrared), sensible and turbulent latent heat exchanges by means of computational fluid dynamics between urban surfaces, plants and the atmosphere^75^ and has been validated in several projects^76,77^. The simulation domain had 244 × 166 × 24 (x–y–z) cells with sizes of 6 m, 6 m, and 0.5 m, respectively. The mesh in the vertical direction was finer near the ground, allowing a better accuracy for edge effects, with a telescoping grid increase factor of 20%. The telescoping effect allows the highest building height of 80 m in the model to be obtained. The height of the top of the 3D model was 179.68 m, with sufficient space above the model as recommended^78^. The model had a mix of residential and commercial land uses covering an area of over 1 km^2^ (width 780 m and length 1,390 m). Floor area ratio in the simulation domain is about 1.3. The model was forced with data out of the simulation domain (Darwin airport weather station^61^), providing the boundary conditions. We modeled the unmitigated scenarios with the relevant albedo of buildings and urban surfaces typical of built environments (Supplementary Table 5). Then, we simulated different mitigation scenarios with the description given below:

- G_20%_ and G_30%_: increased greenery to occupy 20% and 30% of the open spaces with a plantation of grass and additional 260 and 477 mature trees with very dense foliage (Height: 15 m; Albedo: 0.2; Transmittance: 0.3), respectively. Trees and plants were placed in open spaces creating small, medium, and large green spaces;
- ALB_0.4_ and ALB_0.6_: the global-scale (city-scale) application of cool materials, yielding an increase in albedo of all pavements and roofs in the city from 0.2 to 0.4 and 0.6, respectively;
- W: the application of 10 water spray systems at a height of 4 m in the city center commercial district (Droplet diameter: 10 µm; Droplet density: 1 g/cm^3^; Emission rate: 5 µ/s);
- CP: the application of cool materials, i.e., reflective pavements, with an albedo of 0.5;
- SH: providing solar control and shading on top of the main streets of Darwin;
- GR: the use of green roofs in all buildings by planting grass as roof top greening;
- CR: the application of cool materials, i.e., reflective roofs with an albedo of 0.85;
- ALB_SH_G: the combination of a precinct-scale increase in albedo to 0.6, application of shading on streets and parking lots, and 30% increased greenery;
- ALB_SH_G_W: the combination of a precinct-scale increase in albedo to 0.6, application of shading on streets and parking lots, 30% increased greenery, and the water spray systems.

We assessed the prevailing summertime synoptic conditions (northwesterly wind direction with a speed of 5 m/s) in the unmitigated and mitigated scenarios using the same boundary conditions. In total, 48 simulations were performed for the northwesterly and southeasterly wind directions with wind speed values of 5 m/s and 1 m/s. These synoptic conditions correspond to the highest and lowest average ambient temperatures. We discuss the model outputs for the southeasterly synoptic conditions for comparison. For the purpose of analysis, the microclimate simulations were performed for a typical warm day (20th February 2016) in wet season and a representative day in dry season (24th June).

### Validation of the model

Supplementary Table 6 summarizes the settings for each simulation ran for the purpose of validation during different seasons with dry and wet surface conditions. In this study, we used coefficient of determination (R^2^), root-mean-square error (RMSE) and mean average error (MAE) as measures of overall model fit. RMSE helps to capture the differences in absolute values between the simulated and measured data, while R^2^ determine the strength of relationships between variables.

We simulated the unmitigated scenario based on the conditions in the three-day monitoring campaign from 07:00 for 12 h using climate data obtained from Darwin airport weather station^61^. The predicted T_s_ was close to the spatial distribution of T_s_ measured in the ground-based and aerial monitoring (Supplementary Table 7 and Figure 7 (a and b)). The coefficient of determination (R^2^) between the simulation and measurement T_s_ was 0.95.

To compare simulated and measured T_a_, the experimental data obtained from the network of 14 temperature sensors were used to validate the microclimate model of the city of Darwin. We simulated the unmitigated scenario with the boundary condition of three days during the long-term monitoring campaign. We employed semi-hourly meteorological data from the BoM weather station to generate the forcing file for the simulation and adjusted the solar radiation by a factor of 0.90 to match the solar radiation data. We ran the simulation for 46 h for the selected days in wet season starting three hours before sunrise (03:00, 18–19 November 2017) with updated surface data every 30 s. Then, we used the model output data from 06:00 for analysis. We also ran the simulation for a day in dry season (14 May 2018) to evaluate the model T_a_ performance in a different season. Simulations were performed for 15 h and model outputs from 8:00 to 14:00 were compared with the observed T_a_. For the regression analysis, the modeled and observed values were compared independently for each urban weather station. The evaluation of the model performance using correlation and difference measures are tabulated in Supplementary Table 8.

Supplementary Figure 7 (c and e) depicts the simulation outputs (T_a_) plotted against the data measured in Darwin city during wet and dry seasons. The regression analysis between the measured and simulated T_a_ values suggests that the ENVI-met model reproduces the measured data and the major features of the temporal and spatial distributions of the near-ground air temperature in the city. The obtained R^2^ between the simulation and measurement results was 0.9 (wet season) and 0.8 (dry season). When measured and simulated T_a_ for all urban stations are compared, RMSE was 1.24 °C and 1.20 °C for wet and dry seasons, respectively. The RMSE between the model estimations and the actual measurements during the wet season varied from 0.93to 1.54 °C and MAE was between 0.83 and 1.34 °C when data from individual stations were used.

The hourly average T_a_ (average of all urban stations) is shown for the simulated and observed data (Supplementary Figure 7 (d and f)). The mean discrepancy between the average measured and simulated T_a_ over 43 h of the selected days in wet season is 0.97 °C (Standard Deviation = 0.5), and 0.88 (Standard Deviation = 0.5) in dry season. The model underestimates measurement at night, which is explained by the initialized boundary condition of the airport. This is clear from the graph showing spatially averaged T_a_. The ENVI-met model performance can be enhanced when locally measured initialization parameters are used^80^; however, we constructed ENVI-met model according to the actual geometry of the city and initial boundary condition based on the data obtained from the airport due to lack of available meteorological data in the city of Darwin.

The simulated data show generally a good agreement with observed meteorological station data, which was apparent with both R^2^ and difference indices. The slight discrepancy in the simulated data and the corresponding errors can be explained by (a) the anthropogenic heat flux resulting from the use of air conditioners, transportation and other human activities that cannot be taken into account in the model and (b) inaccuracies in the simulation inputs for surface materials, soil, vegetation conditions, and solar radiation. The stations placed at the northern boundaries of the model reveal slightly higher temperatures in the simulations than those in the observations because of the model edge, which performs as an entry door to the warm northeasterly winds. Our model was deemed reliable for microclimate simulation with a performance similar to other studies in tropical cities^79,80^ considering that this study aims to define relative quantities such as air temperature reductions rather than absolute values.

### Impacts of mitigation

The three mitigation strategies of urban greenery (G_30%_); use of cool materials (ALB_0.6_); and the combined scenario of cool pavements, shading, and greenery (ALB_SH_G) were selected.

#### Calculation of building energy needs

We used dynamic building energy simulations (BESs) to examine the energy savings of two buildings representative of Australian building stocks for low-rise dwellings and offices with the application of mitigation strategies. We computed the multizone building energy balance with EnergyPlus as a BES engine^57^, using 2016–2017 weather data. The modeled residential building is a brick veneer single-story building with a total floor area of 218 m^2^. The office building has three stories with a total floor area of 4,840 m^2^. We used typical building models and simulation settings, which are provided in Supplementary Figure 8 and Table 9. According to Geoscience Australia – NEXIS^60^, majority of residential buildings in Darwin LGA are detached houses with a metal sheeting roof. The average building area is 246 m^2^ and 91% of buildings were built before 1980. Further, 40% of commercial buildings in Darwin LGA have 1–3 stories and mostly built before 1980s. Thus, most of the existing building stock were built before the introduction of the energy conservation measures in National Construction Code. The two buildings are representative of the non-retrofitted building stock, which demonstrates a large fraction of existing building stock in Darwin LGA.

We calculated the total, sensible, and latent cooling needs for residential and office buildings on an annual and seasonal basis using modified weather data for the mitigation scenarios. For the mitigated scenarios, the ambient temperature in the weather files for the mitigated scenarios is reduced by the different mitigation technologies whose effect was simulated with the microclimate model, the relative humidity recomputed at the lower temperature with the same specific humidity and all the other weather parameters unchanged. We computed the cumulative cooling energy savings for the LGA based on the total floor area of residential and commercial buildings in the city of Darwin.

#### Electricity demand statistical information

To quantify the impact of the local climate mitigation strategies on the electricity demand, we developed a model with the measured electricity data for the unmitigated condition (see Supplementary Figure 2). We derived the relation between ambient conditions and electricity demand and thus the impact of local climate mitigation strategies. We computed a correlation between the urban environmental parameters and semi-hourly electricity demand using the Eureqa tool for the dry and wet periods^49^. Considering the rapid increase in population in recent years, the variation in the metering system and efficiency, and the small size of the city with a large number of visitors, the period from February 2016 to December 2017 was analyzed. We developed a parametrization that provides the average urban temperature (T_a,urb_) as a function of the airport air temperature (T_a,airport_), global horizontal irradiance (GHI), and wind speed (U) as follows:

1 $${\text{T}}_{{{\text{a}},{\text{urb}}}} = {1}0.{21} + 0.{675} \times {\text{sma}}\left( {{\text{T}}_{{{\text{a}},{\text{airport}}}} ,{ 3}} \right) + 0.00{2} \times {\text{GHI}} + 0.0{55} \times {\text{T}}_{{{\text{a}},{\text{airport}}}} \times {\text{sma}}\left( {{\text{U}},{ 7}} \right) - {1}.{729} \times {\text{sma}}\left( {{\text{U}},{ 7}} \right) + {1}.{\text{832e}} - {6} \times {\text{GHI}}^{{2}}$$

where *sma*(*x*, *n*) is the simple moving average of the previous *n* records of the quantity *x*. The surrogate model shows an R^2^ of 0.93; a median absolute error of 0.3 ºC; and for 99% of the values, the error is less than 2 °C (Supplementary Figure 9).

There were some outliers due to thunderstorms that reached the urban area and the airport inducing a sudden temperature drop. The surrogate model can reproduce the trend and the peaks in the urban temperature profile, at times with a time shift of 30–60 min, with most of the discrepancies occurring during nighttime. We derived a surrogate model to compute the semihourly electricity demand (EL_Dem_) in MVA as a function of T_a,airport_, dew point temperature (T_d_), and GHI (Supplementary Figure 10) as follows:

2 $${\text{EL}}_{{{\rm Dem}}} = {3}.0{24} + 0.{897} \times {\text{sma}}\left( {{\text{T}}_{{{\rm a},{\text{airport}}}} ,{ 54}} \right) + 0.000{3}.\,{\text{T}}_{{{\rm d}}} \times {\text{sma}}\left( {{\text{GHI}},{ 3}} \right) - {11}0{8}/(107.058 + 0.{3116} \times {\text{delay}}\left( {{\text{GHI}},{285}} \right) + 0.0{326} \times {\text{GHI}} \times {\text{delay}}\left( {{\text{GHI}},{ 285}} \right) + {\text{sma}}\left( {{\text{GHI}},{ 12}} \right)$$

where *sma*(*x*, *n*) is the simple moving average of the previous *n* records of the quantity *x*. *Delay*(*x*,* n*) represents a delayed variable; namely, for time step *t*, it considers its value *n* time steps before.

Equation (1) was used to compute the mitigated temperatures translated to the airport. We used this inverted approach looking for a correlation between electricity and airport data to minimize error propagation, considering the performance during the training of the model. We developed the correlation given in Eq. (1) instead of using measured urban ambient temperatures since the urban temperature data have been collected for one year (from September 2017) while the electricity demand data relate to an earlier and longer period (February 2016–December 2017). Therefore, a transformation function was necessary.

Based on the results of the surrogate model, humidity has a nonlinear impact on the demand, affecting the performance of the parametrization in the intermediate temperature range, while the demand is monotonic with temperature (Supplementary Figure 10). The performance of the surrogate model (R^2^ = 0.79) is comparable to that of Thatcher^42^, with an r-squared value equal to 0.82. The surrogate model captures the fuzziness in the demand but shows a strong influence on the humidity level, which explains the peak electricity demand that occurs for both hot and humid conditions (Supplementary Figure 11).

#### Heat-related health statistical information

We calculated the heat-related mortality and morbidity for the mitigation scenarios using modified half-hourly temperatures based on the simulation results. Half-hourly temperatures and RH values were obtained from Darwin BoM airport station between January 2002 and June 2017 and validated using the method described above. The all-cause mortality data (between 2000 and 2015) and the daily hospital admission data considering only heat-relevant diagnoses (between July 2000 and June 2017) were provided by the Northern Territory Government Department of Health. We classified hospital admissions as ‘heat-relevant’ based on previous studies^81,82^.

To estimate the improvement in the whole population’s heat-related health, we developed the relationship between the weather parameters and daily mortality and morbidity rates. To account for longitudinal, seasonal, and cyclic changes, the time series of mortality and morbidity data were decomposed using exponential smoothing and Fourier series. Regression analysis was adopted to assess and describe the relationship between two variables.

Due to a lack of cold-related health issues^81^, any temperature-related anomalies in morbidity and mortality can be defined as heat-related. The analysis showed strong relationships between the daily anomalies in hospital admissions within the Darwin urban health district and both the daily maximum and mean temperatures between 2002 and 2017 (*p* = 0.00). An increase of 1 °C in the daily maximum temperature increases hospital admissions by 7.9%, while a 1 °C rise in the daily mean temperature elevates that by 6.6%. When the dataset was filtered for days with higher temperatures and RH levels, the strongest significant relations were found on days with both daily mean temperatures and RH levels above the 80th percentiles calculated for the whole period. On these hot days with high humidity, a 1 °C increase in the daily maximum temperature raised the number of hospital admissions by 263%.

We analyzed the relationship between the daily anomalies in deaths and the weather parameters using the same method^18,83^. The regression analysis (Supplementary Figure 12) showed relationships between both the medians and averages of the daily anomalies in deaths and daily maximum temperatures binned in a 1 °C increase (adjusted *R*^2^ = 0.46, *p* = 0.005 and adjusted *R*^2^ = 0.32, *p* = 0.021, respectively). An increase of 1 °C in daily maximum temperatures raises the medians and averages of the mortality anomalies by 4.9 and 4.4%, respectively.

The analysis was repeated (Supplementary Figure 13) for the mortality data series aggregated for the Darwin urban and rural health districts (adjusted *R*^2^ = 0.50, *p* = 0.003 and adjusted *R*^2^ = 0.33, *p* = 0.018, respectively). An increase of 1 °C in daily maximum temperatures increases the medians and averages of the mortality anomalies for the whole urban and rural regions by 6.4% and 5.3%, respectively.

Daily mortality data for the age group above 65 were not available for confidentiality reasons. Therefore, we used the model of Woodruff et al.^81,82^ corresponding to heat-related mortality of people aged over 65 years.

Both our new models described above and the model from Woodruff et al.^81,82^ were used to calculate the impact of mitigations on heat-related health for the whole and the oldest populations in Darwin.

## Supplementary information

Supplementary information.
