# Supplementary material for: Holistic approach to assess co-benefits of local climate mitigation in a hot humid region of Australia
Source: Sci Rep. 2020 Aug 26;10:14216. doi: 10.1038/s41598-020-71148-x (PMC7450084; doi:10.1038/s41598-020-71148-x)
Supplement: Supplementary file 1 — Supplementary information. [file 41598_2020_71148_MOESM1_ESM.pdf]

Supplementary Information

Holistic approach to assess co-benefits of local climate mitigation in a hot humid region of Australia

Shamila Haddad[1], Riccardo Paolini[1], Giulia Ulpiani[1], Afroditi Synnefa[1], Gertrud Hatvani-Kovacs[1], Samira Garshasbi[1], Jonathan Fox[1], Konstantina Vasilakopoulou[1], Lawrence Nield[2], and Mattheos Santamouris[1,*]

[1]The University of New South Wales, UNSW Built Environment, Sydney, Australia

[2] Former Northern Territory Government Architect, Darwin, Austraila

Correspondence to: m.santamouris@unsw.edu.au

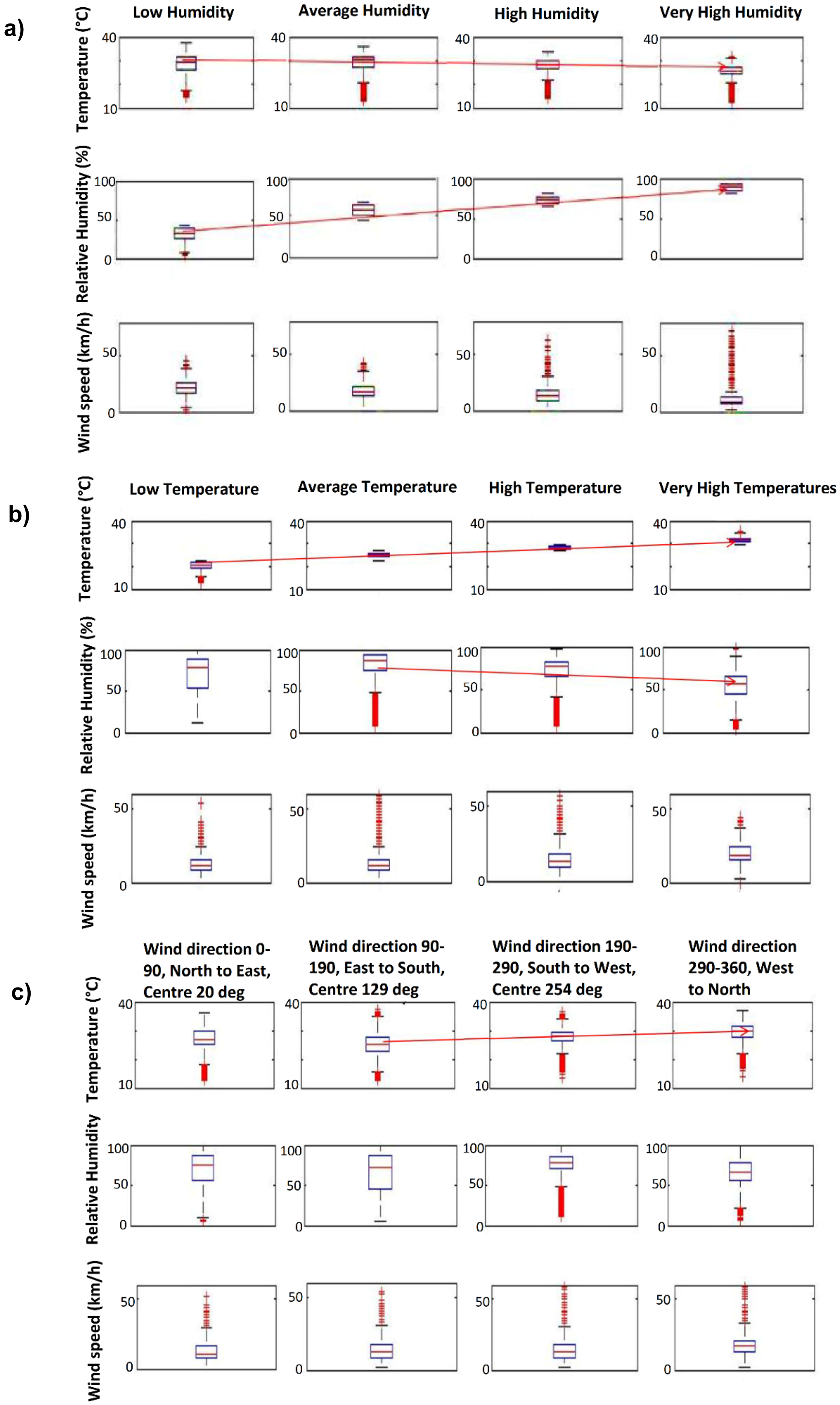

Figure S1. Clustering results according to the humidity (a), temperature (b), wind direction (c)

Measurement locations: The monitoring campaign covers a land area of over 1 km^2 (Supplementary Figure 2). During long-term monitoring, T_a was recorded every 30 min in 15 locations for approximately one year (2017-2108). The three-day monitoring campaign (2016) included 20 measurement points to characterize the city (Supplementary Figure 2). We collected data at each point over 10 min with a time step of one minute. The main outputs of the monitoring activity are split into the following categories:

- *Interparameter comparative assessment in absolute terms*: We analyzed the trends of air temperature, RH, wind speed and net radiation for every point of measurement to characterize the local thermal behavior.
- *Interpoint comparative assessment in absolute terms*: We calculated the average air temperature (ΔT_a) and RH (ΔRH) within measurement points that were close in space and time (within one hour). The intermittent occurrence of short torrential rain and scattered thundershowers reduced the number of suitable data for the comparison, while indicating other facets of the local microclimate. These include the increase in the moisture content and the ambient temperature drop after a storm. The RH increased up to 16.5%, and the ambient temperature decreased by 4.52 °C on average. The transient thermal behavior of asphalt surfaces after a rainfall shows that the surface temperature exhibited an increase of even more than 1 °C in the 10-min time slot of measurement: the peak value for streets was 2.49 °C, while the corresponding value for parking lots was 1.7 °C. The average duration and magnitude of rainfall impel the formation of a quite thin film of water over hot asphalt surfaces of streets and pavements.
- *Interpoint comparative assessment in relative terms:* We calculated the differences between the data recorded simultaneously in Darwin city and the airport BoM station.

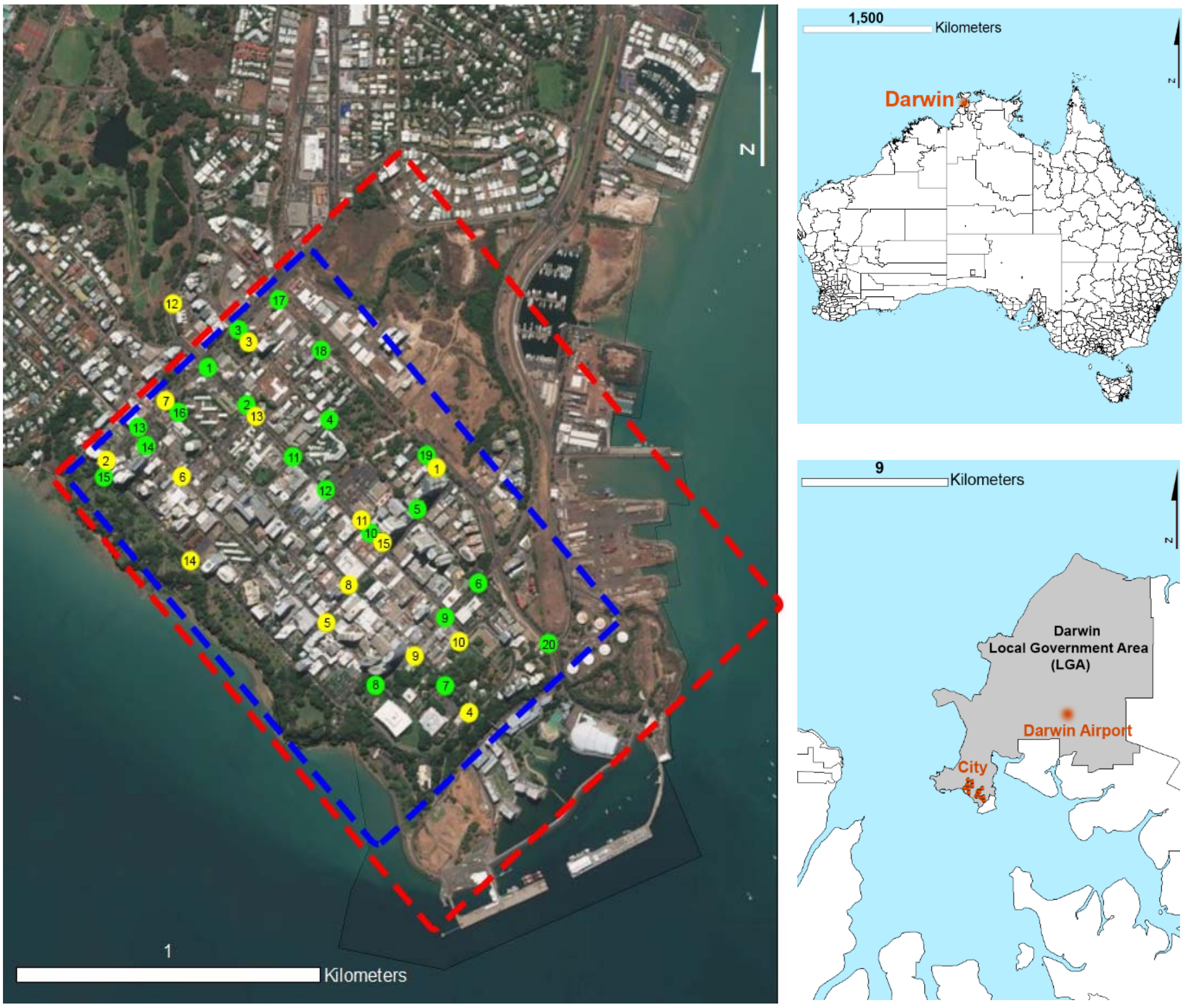

Figure S2. Map of the study area. Locations of measurement points in the three-day ground-based survey (green) and the long-term ground-based monitoring survey (yellow), selected area relative to the analyzed portion of the grid in the electricity data analysis (red), microclimate simulation domain (blue). (Maps are generated using ESRI ArcGIS 10.6: www.esri.com).

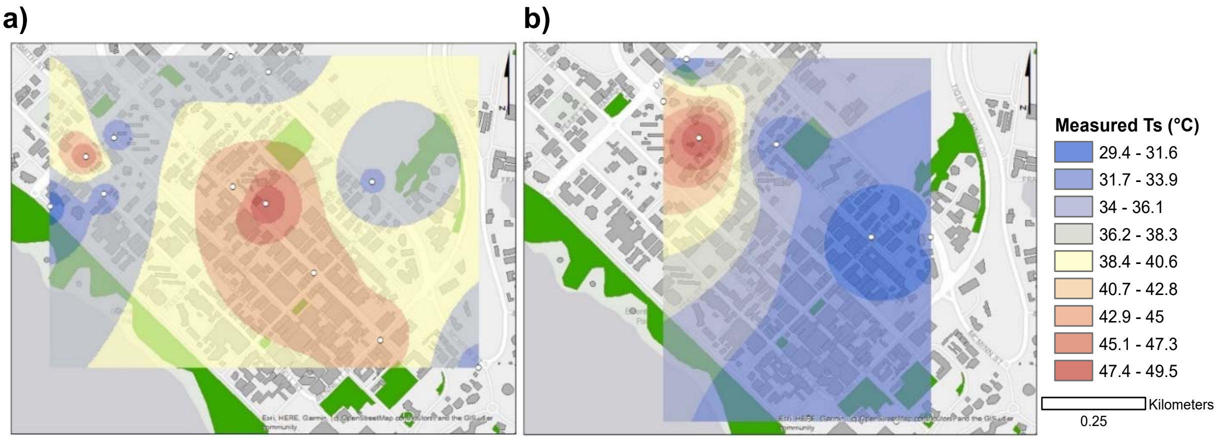

Figure S3. Spatial distribution of T_s recorded during short-term ground-based monitoring; 24th March 2017 (a), 25th March 2017 (b). (Maps are generated using ESRI ArcGIS 10.6: www.esri.com).

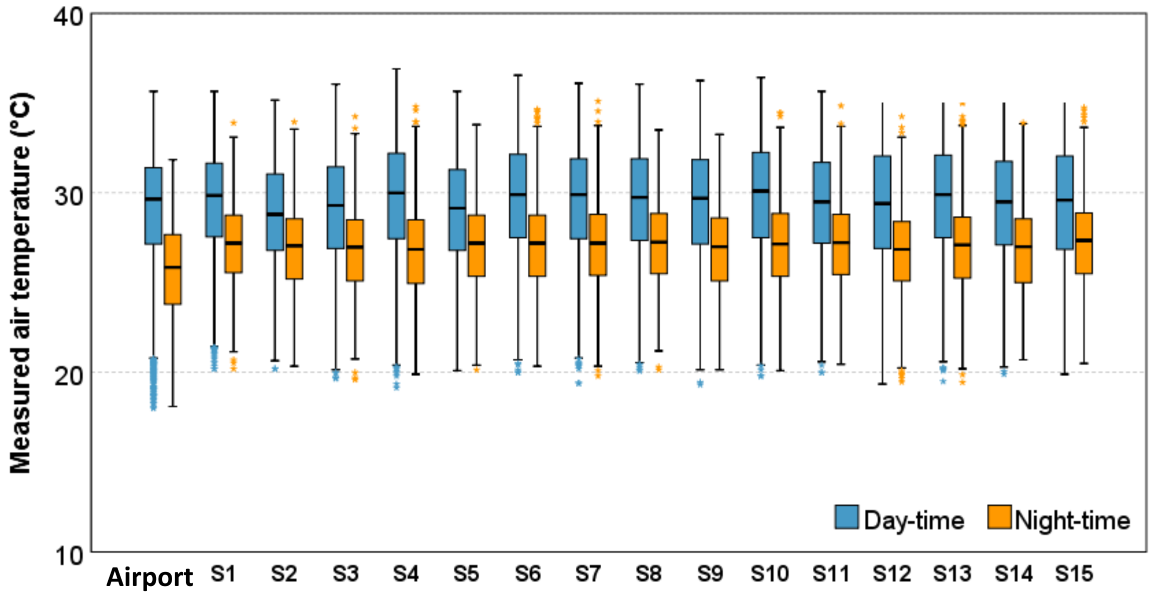

Figure S4. Box plots of the nighttime and daytime hourly temperature per measurement point

a)

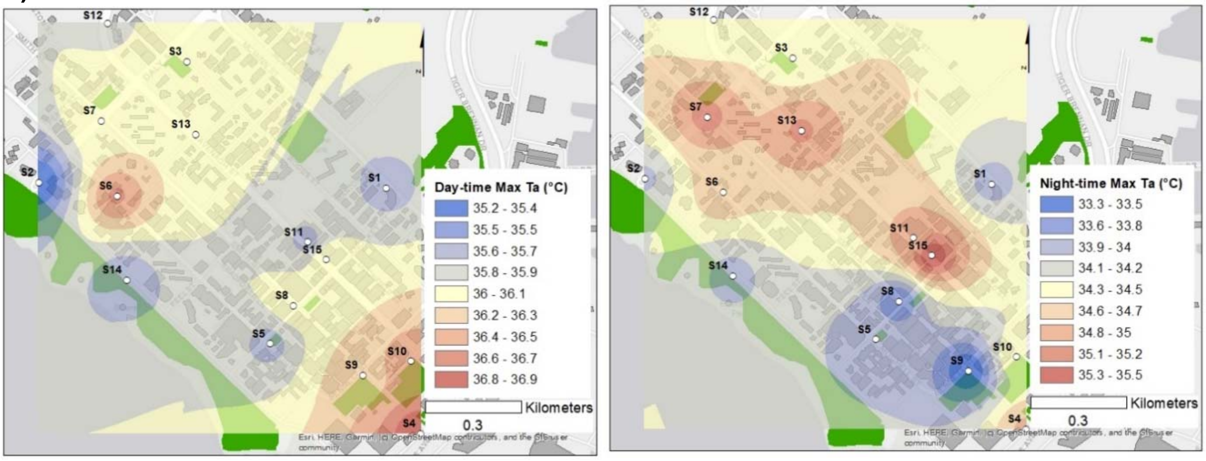

b)

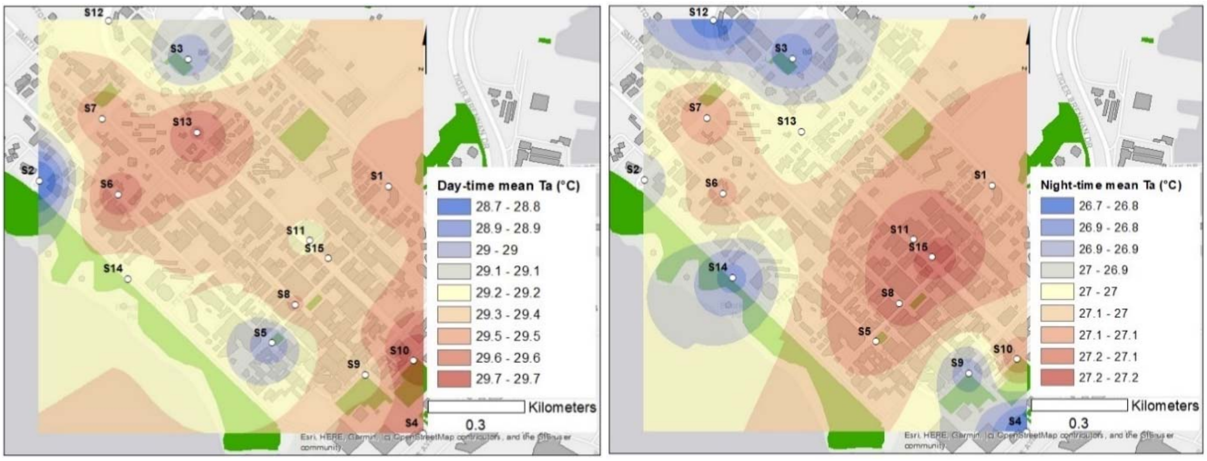

Figure S5. Spatial distribution of daytime and nighttime average T_a (a) and daytime and nighttime maximum T_a (b) recorded during long-term monitoring. (Maps are generated using ESRI ArcGIS 10.6: www.esri.com).

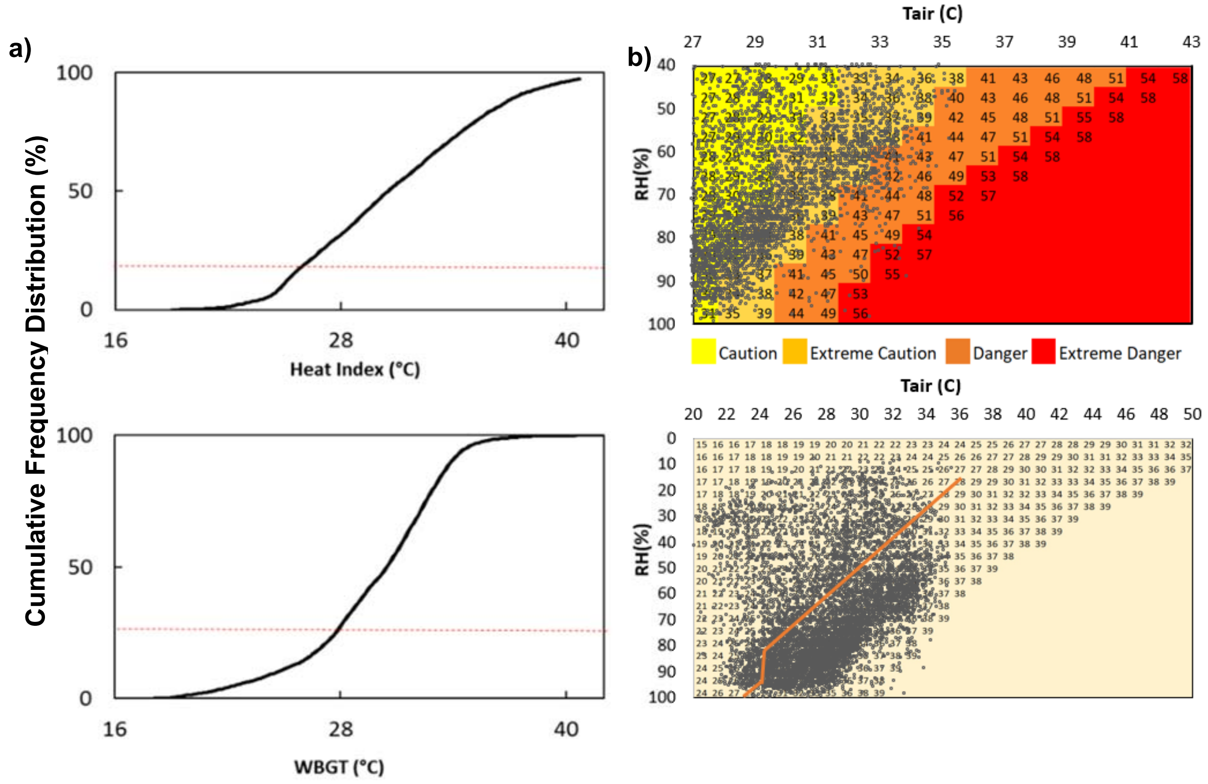

Figure S6. Cumulative frequency distribution of the Heat Index and WBGT (a), Scatter plot of temperature and humidity on the Heat Index matrix from National Weather Service (top) and WBGT matrix obtained from Australian Bureau of Meteorology (bottom) (b)

Table S1. Technical specifications of the Aerial infrared monitoring

**Monitoring equipment**	**Specifications**
**Drone - DJI INSPIRE 1**	
Model	T600
Weight	6.27 lbs (2845 g, including propellers and battery, without gimbal and camera)<br>6.76 lbs (3090 g, including propellers, battery and Zenmuse XT)
GPS Hovering Accuracy	Vertical: ±1.64 feet (0.5 m)<br>Horizontal: ±8.20 feet (2.5 m)
Max Speed	49 mph or 79 kph (ATTI mode, no wind)
Max Flight Time	Approx. 18 min
Battery Type	LiPo 6S
**Thermal camera - ZENMUSE XT**	
FPA/Digital Video Display Formats	640 × 512<br>336 × 256
Pixel Pitch	17 µm
Spectral Band	7.5 - 13.5 µm
Full Frame Rates	640 × 512 ：30 Hz (NTSC) 25 Hz (PAL)<br>336 × 256 ：30 Hz (NTSC) 25 Hz (PAL)
Sensitivity (NEdT)	<50 mK at f/1.0
Scene Range (High Gain)	640 × 512 ：-13° to 275°F (-25° to 135°C)<br>336 × 256 ：-13° to 212°F (-25° to 100°C)
Scene Range (Low Gain)	-40° to 1022°F (-40° to 550°C)
Accuracy (Radiometric)	±10 °C in any part of the image.<br>±5 °C in ideal conditions.
Spot Meter	Temperatures measured in central 4×4
Operating Temperature Range	14° to 104°F (-10° to 40°C)
Humidity	5% to 95%

Table S2. Technical specifications of the monitoring equipment-local weather stations

Monitoring equipment	Specifications
**LogTag TRIX-16**	
Accuracy and Resolution	±0.5°C for temperature between -20°C and +40°C and ±0.7°C for temperature above +40°C; 0.1°C

Table S3. Technical specifications of the ground-based measurements equipment

Monitoring equipment	Specifications
**Net radiometer Hukseflux NR01**	
Product type	4-component net radiometer
Included sensors	2 x identical ISO 9060 second class pyranometer & 2 x identical pyrgeometer with 150 ° field of view angle
IR01 pyrgeometer	
Spectral range	4.5 to 40 x 10-6 m
Solar offset	< 15 W/m^2 (at 1000 W/m^2 global horizontal irradiance on the window)
Field of view angle	150 °
Response time (95 %)	18 s
Sensitivity (nominal)	15 x 10-6 V/(W/m^2)
Measurement range	-300 to +300 W/m^2
Spectral range solar	285 to 3000 x 10-9 m
Spectral range longwave	4.5 to 40 x 10-6 m
SR01 pyranometer	
ISO classification (ISO 9060: 1990)	second class pyranometer
Response time (95 %)	18 s
Measurement range	-300 to +300 W/m^2
**MetPak Pro meteorological station**	
Wind measurement	
Parameters	Wind speed and direction
Wind Speed Range	0-60m/s
Wind Speed Accuracy	±2% at 12m/s
Wind Speed Resolution	0.01m/s
Wind Direction Range	0 to 359º - No dead band
Wind Direction Accuracy	±3º at 12m/s
Wind Direction Resolution	1º
Air temperature measurement	
Air Temperature	Pt100 1/3 Class B
Range	-50ºC to +100ºC
Accuracy and Resolution	±0.1ºC; 0.1ºC (0.1ºF)
Barometric pressure measurement	
Range	600-1100hPa
Accuracy and Resolution	±0.5hPa; 0.1hPa
Compensated for temperature dependency	-30ºC to +70ºC
Dew point measurement	
Accuracy and Resolution	±0.15ºC (23ºC ambient temp at 20ºC dew point); 0.1ºC (0.1ºF)

Table S4. Physical characteristics of the city of Darwin and Darwin LGA based on the data obtained from the city of Darwin Open Data Hub

**City of Darwin**		
Tree cover area (pre-cyclone Marcus -2018) (CBD)	452164	m^2
Tree cover percentage	16.6 %	%
Unshaded blacktop bitumen percentage	35%	%
Building footprint (residential and commercial) percentage	13.6%	%
Building footprint (residential and commercial)[1]	369534	m^2
Shade cloth and metal shading structure	2068	m^2
Pathways (footpaths, driveways, and shared paths)	88223	m^2
Asphalt (2.7 %), Concrete (41.7%), Exposed Aggregate (48.2%), No Surface (0.5%), Pavers (6.9%)		
Parking and Carparks	168429	m^2
Road network length	437591	m
Major Parks	163361	m^2
Parklands	285300	m^2
CBD area	2722983	m^2
**Local Government Area (LGA)**		
Tree cover area (pre-cyclone Marcus)	19950156	m^2
Tree cover percentage	17.8 %	%
Shading structure	24656	m^2
Concrete Slab (9.7%), Metal (26.4%), PVC (0.7%), Shade Cloth (53.5%), Steel (1.4%), Steel and Shade Cloth (8.3%)		
Pathways (footpaths, driveways, and shared paths)	960862	m^2
Asphalt (17.7%), Chipseal (0.8%), Concrete (75.7%), Exposed Aggregate (4.3%), Grass (0.1%), Gravel (0.1%), No Path (0.1%), Pavers (1.3%)		
Parking and Carparks	693208	m^2
Road network length	464708	m
Major Parks	1131423	m^2
Parklands	10637180	m^2
LGA Area	112157067	m^2

Note: Data was obtained from Darwin Open Data Hub (http://open-darwin.opendata.arcgis.com/)

[1]: Data obtained from Geoscience Australia – NEXIS

Microclimate simulations. Supplementary Table 4 shows the boundary conditions and input data for the microclimate simulations of the unmitigated scenario. We obtained hourly averages of T_a and the RH from the Darwin airport weather station. We applied a simple model forcing, which allows dynamically changing the meteorological background values for T_a and the RH within a 24-h cycle.

Table S5. Major input parameters for ENVI-met simulation- unmitigated condition.

**Meteorological inputs**	Air temperature and relative humidity	Semi-hourly data from the Australian Bureau of Meteorology
	Wind Speed in 10 m above ground<br>Wind direction	Semi-hourly data from the Australian Bureau of Meteorology: 5m/s<br>60 (deg) anti-clockwise from North
	Global, horizontal solar and infrared radiation	1-minute solar data from the BoM station: factor of shortwave adjustment=0.9
	Initial Temperature Atmosphere	29.45 °C
	Specific humidity in 2500 m	7 g/kg*
**Plants**	Roughness length at reference point<br>Grass<br>Tree	0.01 m<br>Height 0.2 m, LAD= 0.3*(m^2/m^3) with average density<br>15 m tall very dense foliage, distinct crown layer
**Building Surface**	Wall and roof<br>Pavements (Asphalt)<br>Pavements (Concrete)<br>Loamy soil	Albedo=0.2<br>Albedo=0.05<br>Albedo=0.20<br>Albedo=0.15
**Initial soil temperature****		Upper layer (0-20 cm): 304 K**<br>Middle layer (20-50 cm): 304 K**<br>Deeper layer (below 50 cm): 304 K**

*: The parameters are sourced from the default values of ENVI-met, **: values are derived from 3 hourly synoptic weather observations at the Darwin airport.

Calibration of the model. For the purpose of validation, we ran an ENVI-met simulation imposing the environmental conditions of the selected days during the short-term and long-term monitoring campaigns. The model was considered adequate for the microclimate simulations. The discrepancies between the predictions and observations are likely due to the assumptions in the model and local conditions. However, these discrepancies do not significantly affect the results because the results of the mitigation scenarios are presented relative to the unmitigated scenario.

Table S6. Weather conditions of the selected days for validation with short-term ground based and long-term monitoring.

**Season**	**Date**	**Air temperature**			**Mean relative humidity (%)**	**Mean wind speed (m/s)**
		**Max (°C)**	**Mean (°C)**	**Min (°C)**		
LT-Wet season	18 Nov 2017	35.1	29.5	25.6	69%	3.6
LT-Wet season	19 Nov 2017	34.0	29.4	24.5	72%	3.7
LT-Dry season	14 May 2018	30.6	24.9	19.7	35%	4.3
ST-Wet season	24 March 2017	31.4	27.2	24.8	86%	3.0
ST-Wet season	25 March 2017	29.5	26.3	24.1	90%	2.5

Note: data from the airport was used for the initialization due to lack of available meteorological data in the city of Darwin; Short-term (ST), Long-term (LT)

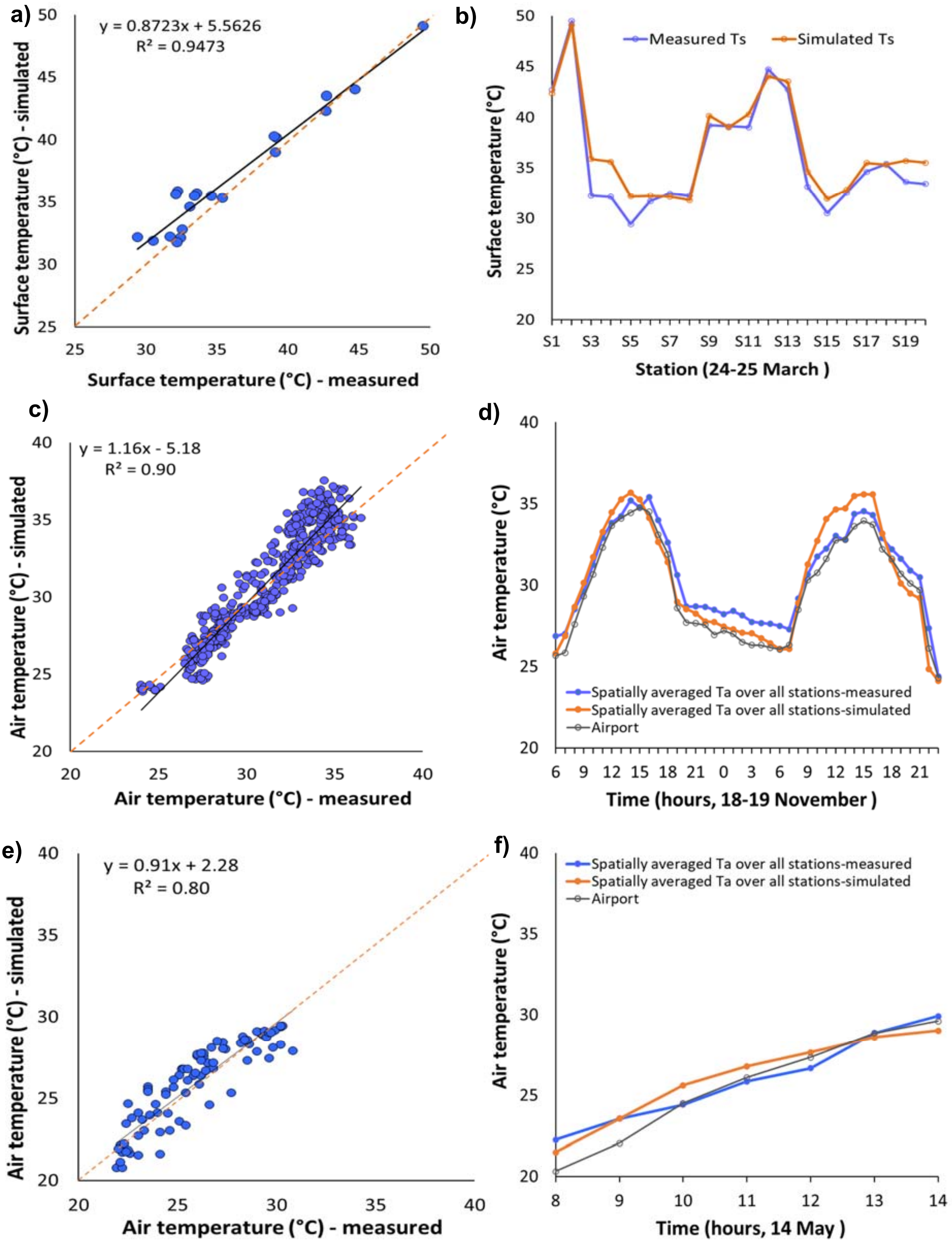

Figure S7. Correlation and comparison between the simulated and measured T_s during short-term monitoring campaign (a, b); Correlation and comparison between the simulated and measured T_a during the long-term monitoring campaign: wet season (c, d), dry season (e,f)

Table S7. Examples of the measured T_s with an overlay of simulated T_s

Surface temperature measurement (aerial monitoring campaign 24 March at 12:00)

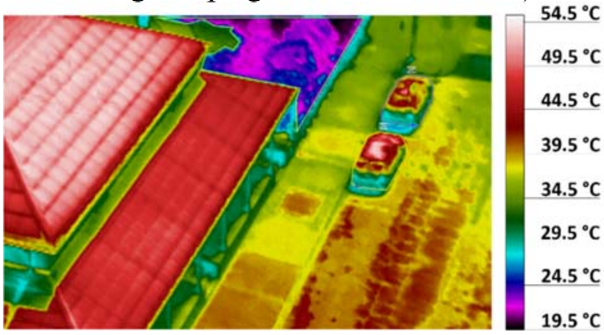

Surface temperature simulated (street and pavement): 30-45.2 °C

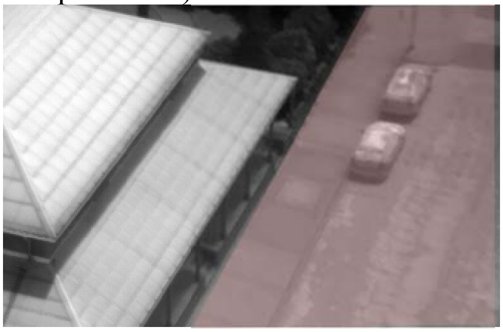

Surface temperature measurement (aerial monitoring campaign 23 March at 15:00)

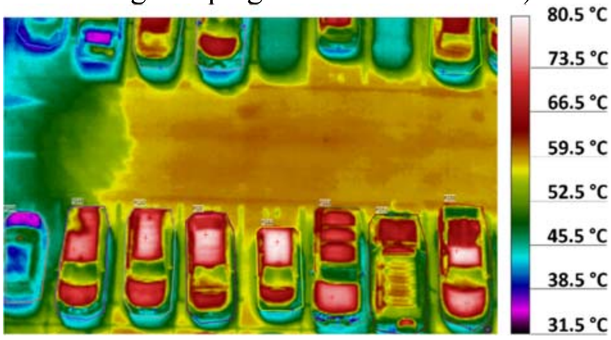

Surface temperature simulated (street and pavement): 45.4- 56.9

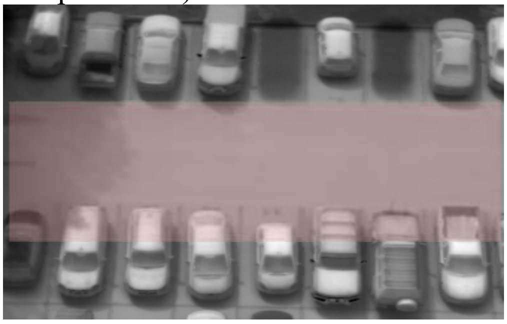

Surface temperature measurement (aerial monitoring campaign 23 March at 15:00)

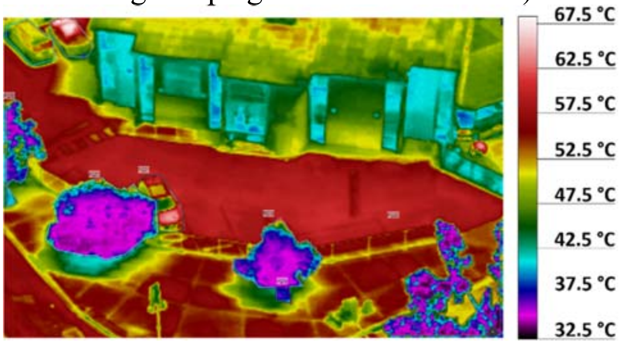

Surface temperature simulated (street and pavement): 47.0-54.4 °C

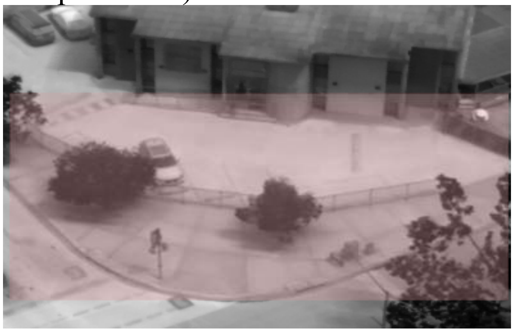

Surface temperature measurement (aerial monitoring campaign 23 March at 11:00)

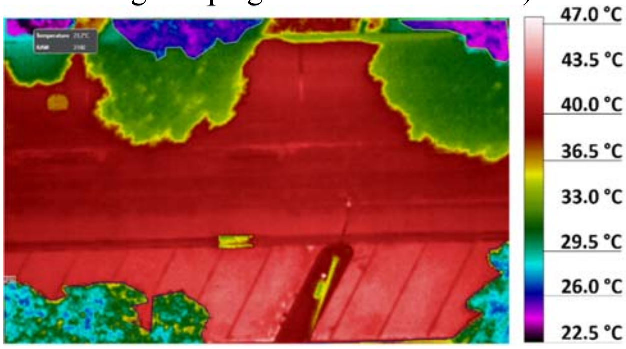

Surface temperature simulated (street and pavement): 28.5-39.7 °C

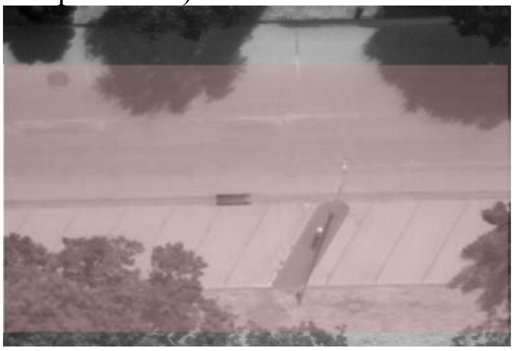

Table S8. Model fit between the simulated and measured daily air temperature (T_a) for all urban stations in the selected days during wet and dry seasons (18-19 November and 14 May 2017): RMSE – root-mean-squared error, MAE – mean average error, R^2 – coefficient of determination

	**Wet season**			**Dry season**		
**Station**	**RMSE**	**MAE**	**R^2**	**RMSE**	**MAE**	**R^2**
All stations	1.24	1.05	0.90	1.20	0.99	0.80
S1	1.40	1.23	0.92	0.85	0.75	0.90
S2	1.54	1.34	0.90	1.28	1.05	0.86
S3	1.25	1.07	0.92	1.53	1.10	0.90
S4	1.20	1.04	0.93	0.74	0.58	0.96
S5	0.93	0.75	0.93	1.24	0.58	0.86
S6	1.54	1.29	0.92	0.73	0.67	0.95
S7	1.42	1.25	0.94	1.06	0.87	0.86
S8	1.09	0.87	0.87	0.88	0.72	0.93
S9	1.29	1.08	0.90	1.18	0.99	0.85
S10	1.14	0.97	0.95	2.26	2.22	0.96
S11	1.12	0.94	0.88	1.20	1.11	0.82
S13	0.96	0.83	0.96	1.22	0.99	0.79
S14	1.05	0.86	0.93	1.35	0.95	0.86
S15	1.18	1.04	0.87	1.01	0.93	0.88

a) b)

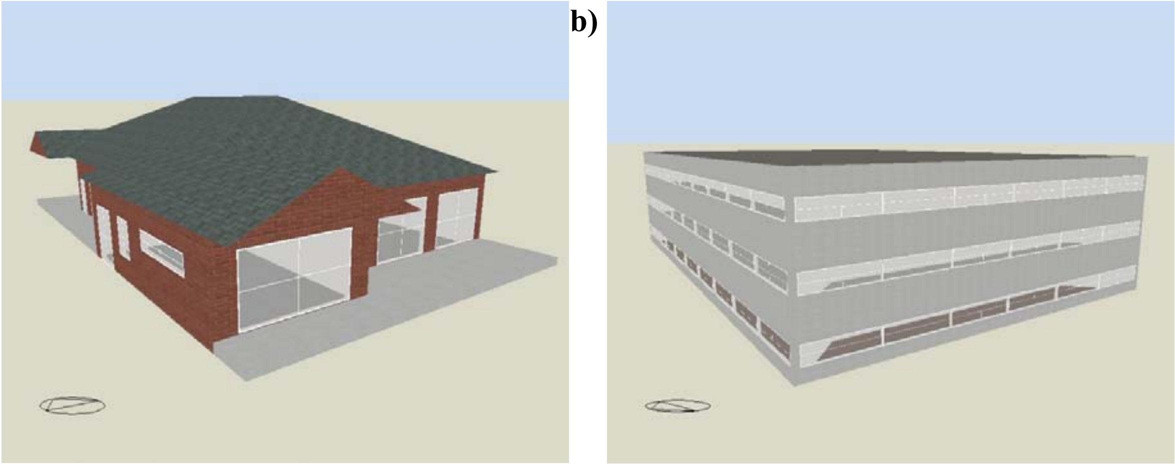

Figure S8. Residential (a) and office (b) building model.

Table S9. Thermal properties of the building envelope and simulation settings

**Building Type**	**Residential Building**	**Office Building**
Roof	Pitched Roof: Light metal, Attic Cavity, Plaster Board, Timber Flooring, Plywood, U-Value = 0.41W/m^{2}K	Built-up roof: Roof membrane, roof insulation, metal decking U value=0.45W/m^{2}K
External wall	Brick veneer U-value = 0.516 W/m^{2}K	Hollow Core Concrete, Gypsum Plastering, EPS Expanded Polystyrene, Gypsum Plasterboard, U-Value=0.683 W/m^{2}K
Ground Floor	Slab-On-Grade Floor, U-Value: 0.276	Slab-On-Grade Floor, U-Value: 0.276
Windows	Glazing: Single Clear 3mm, U-value= 5.89W/m^{2}K, Total Solar Transmission (SHGC): 0.861 Frame: Painted Wooden Frame, U-value: 3.633	Glazing: Single Clear 3mm, U-value= 5.89W/m^{2}K, Total Solar Transmission (SHGC): 0.861 Frame: Painted Wooden Frame, U-value: 3.633
Floor slab	Ceramic and carpet, U-value = 0.4 W/m^{2}K	Ceramic and carpet, U-value = 0.4 W/m^{2}K
Cooling set point temperature (°C)	24	24
Internal gain (W/m^2)	3.58	11.77
Ventilation Rate (ACH)	7	7
Infiltration Rate (ACH)	0.7	0.7
Density (People/m^2)	0.02	0.05

Table S10. Statistical summary of the mitigation results under northwesterly wind direction with the speed of 5m/s

Scenarios	$T_{a, MAX}$ (°C)	$T_{a, MIN}$ (°C)	$\Delta T_{a, MAX}$ (°C)	$\Delta T_{a, MIN}$ (°C)	MAX ΔT_a (°C)
Reference Model	36.4	32.0	-	-	-
Global Albedo $_{0.4}$	34.8	31.9	1.7	0.0	1.7
Global Albedo $_{0.6}$	33.9	31.9	2.5	0.1	2.8
Cool pavements	34.3	31.9	2.1	0.1	2.2
Shading	35.1	31.7	1.3	0.2	1.9
Greenery $_{20\%}$	35.8	32.0	0.6	0.0	2.6
Greenery $_{30\%}$	35.2	31.9	1.2	0.0	2.7
Cool roof	36.3	32.0	0.2	0.0	0.7
Green roof	36.0	31.7	0.5	0.2	1.6
Water fountain	34.5[a]	30.1	2.0[a]	1.9	3.9
Combined	33.7	31.9	2.7	0.1	3.5
Combined & water	33.7	28.9	2.7	3.1	5.2

[a]: $T_{a, MAX}$ and $\Delta T_{a, MAX}$ correspond to the area where the mitigation strategy was employed

Table S11. Statistical summary of T_a for 2016 calendar year and corresponding air temperature difference (ΔT) between the reference case and mitigation scenarios.

	Air temperature. (˚C)				ΔT (˚C)		
	Reference	G30%	$ALB_{0.6}$	ALB_SH_G	G30%	$ALB_{0.6}$	ALB_SH_G
Mean	28.2	27.9	27.6	27.5	0.3	0.6	0.7
Median	28.2	28.0	27.7	27.6	0.3	0.6	0.7
Max	36.6	36.3	35.9	35.7	0.3	0.8	0.9
Min	14.0	13.9	13.8	13.7	0.1	0.2	0.3
99th percentile	34.1	33.8	33.4	33.2	0.3	0.7	0.9
1st percentile	19.2	19.0	18.9	18.8	0.2	0.4	0.4

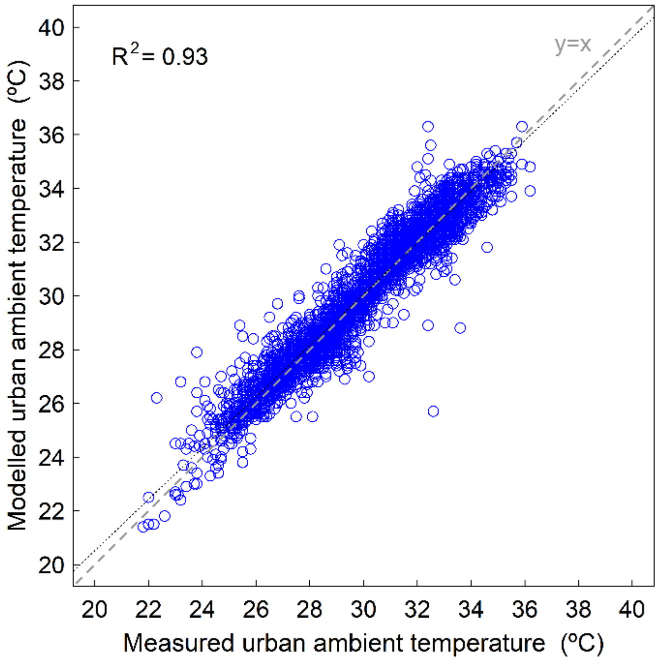

Figure S9. Modelled vs. observed average urban ambient temperature in the city of Darwin.

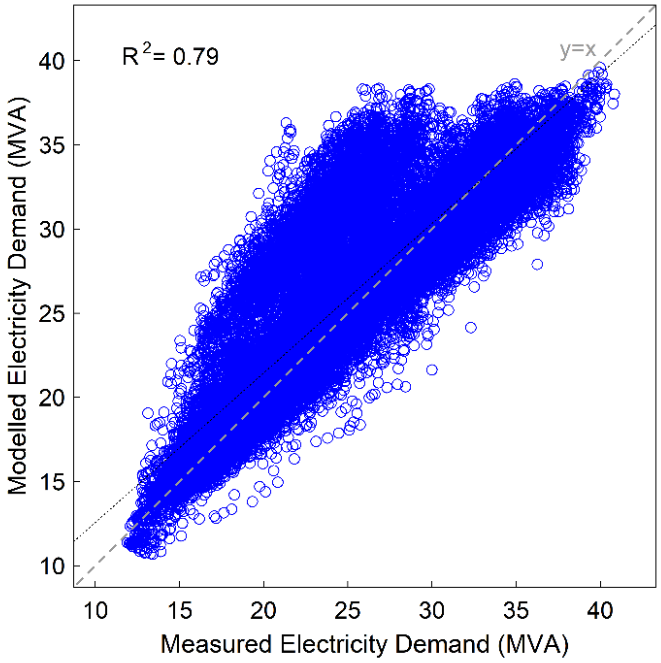

Figure S10. Modelled vs. observed electricity demand (semi-hourly values) for the Darwin city centre and a portion of Frances Bay.

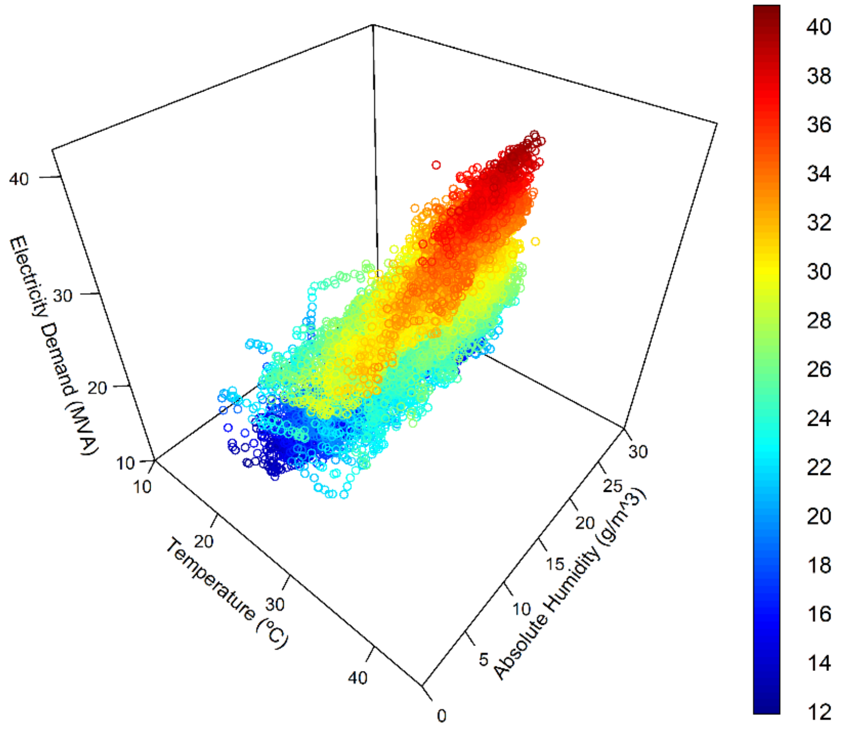

Figure S11. Observed electricity demand vs. the urban ambient temperature and absolute humidity.

Table S12: Statistics for the electricity demand in the unmitigated (observed and modelled) and mitigated scenarios, expressed in MVA.

Stat	Unmit obs (MVA)	Unmit mod (MVA)	Greenery (MVA)	Cool material (MVA)	Combined (MVA)
Max	40.8	39.6	38.9	39.3	38.8
Average	24.2	25.2	24.6	24.9	24.5
Median	23.7	25.7	25.1	25.4	25.0
Min	11.9	10.7	10.3	10.6	10.3
99th percentile	37.5	36.8	36.2	36.5	36.0
1st percentile	14.2	14.3	13.8	14.0	13.7

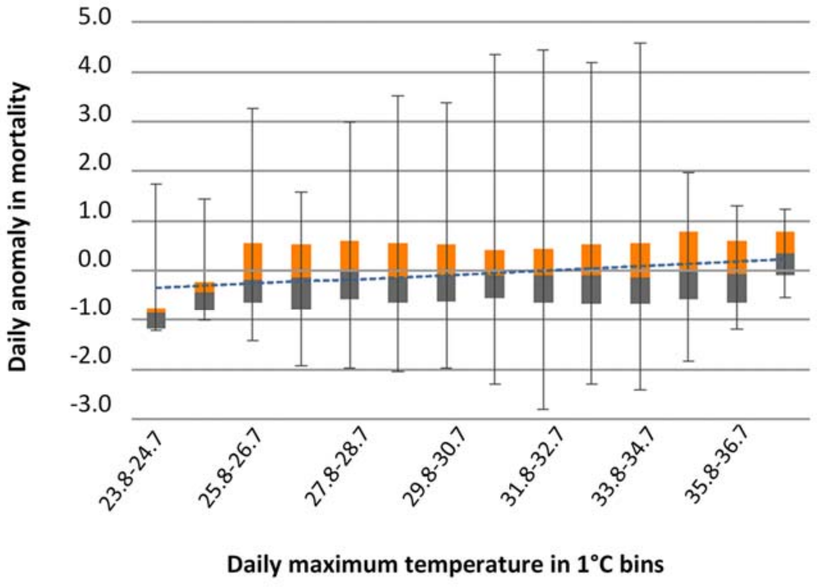

Figure S12. Median, minimum, maximum,25th and 75th percentiles of daily anomalies in mortality grouped at 1°C intervals of daily maximum temperatures in Darwin Urban Health District with the predicted average of daily anomalies in mortality superimposed

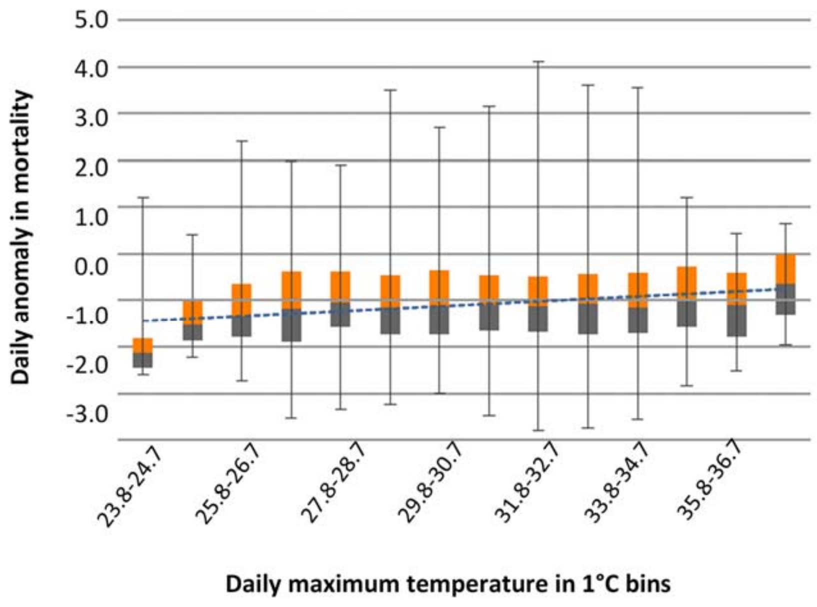

Figure S13. Median, minimum, maximum, 25th and 75th percentiles of daily anomalies in mortality grouped at 1°C intervals of daily maximum temperatures in Darwin Urban and Rural Health Districts with the predicted average of daily anomalies in mortality superimposed
